# An activity-dependent local transport regulation via degradation and synthesis of KIF17 underlying cognitive flexibility

**DOI:** 10.1126/sciadv.abc8355

**Published:** 2020-12-16

**Authors:** Suguru Iwata, Momo Morikawa, Yosuke Takei, Nobutaka Hirokawa

**Affiliations:** 1 Department of Cell Biology and Anatomy, Graduate School of Medicine, The University of Tokyo, Bunkyo-ku, Tokyo 113-0033, Japan.; 2 Laboratory for Molecular Psychiatry, RIKEN Center for Brain Science, Saitama 351-0198, Japan.; 3 Department of Anatomy and Neuroscience, Faculty of Medicine, University of Tsukuba, Tsukuba 305-8577, Japan.

## Abstract

Synaptic weight changes among postsynaptic densities within a single dendrite are regulated by the balance between localized protein degradation and synthesis. However, the molecular mechanism via these opposing regulatory processes is still elusive. Here, we showed that the molecular motor KIF17 was locally degraded and synthesized in an *N*-methyl-d-aspartate receptor (NMDAR)–mediated activity–dependent manner. Accompanied by the degradation of KIF17, its transport was temporarily dampened in dendrites. We also observed that activity-dependent local KIF17 synthesis driven by its 3′ untranslated region (3′UTR) occurred at dendritic shafts, and the newly synthesized KIF17 moved along the dendrites. Furthermore, hippocampus-specific deletion of *Kif17* 3′UTR disrupted KIF17 synthesis induced by fear memory retrieval, leading to impairment in extinction of fear memory. These results indicate that the regulation of the KIF17 transport is driven by the single dendrite–restricted cycle of degradation and synthesis that underlies cognitive flexibility.

## Teaser

Neuronal activity–dependent dampening and regeneration of dendritic KIF17 transport underlie cognitive flexibility.

## INTRODUCTION

Multiple neuronal functions require decentralized processes including activity-dependent localized protein degradation and synthesis. The dendritic remodeling induced by the balance between these opposing regulatory processes is independent of the molecular mechanism involving the longer time scale nuclear transcriptional regulation (*1*). The ubiquitin-proteasome system (UPS), by which the proteasome recognizes and degrades targeted polyubiquitinated proteins, is involved in localized protein degradation and hence regulates synaptic functions (*2*–*5*). Dysregulation of the UPS by mutating a specific ubiquitin ligase or injecting proteasome inhibitors into the hippocampal CA1 causes impairments in long-term potentiation (LTP) and hippocampal-dependent memory tasks (*6*–*8*). In particular, the *N*-methyl-d-aspartate receptor (NMDAR)–mediated UPS has been reported to be involved in the degradation of various kinds of synaptic proteins, such as scaffolding proteins and glutamate receptors, in dendrites (*9*). The UPS not only removes proteins from the postsynaptic density but also influences various signaling cascades, which leads to the remodeling of synapses (*10*, *11*).

Local control of protein production could be needed to maintain the various forms of plasticity that require de novo protein synthesis (*12*, *13*). Neuronal stimulation activates several signaling pathways that control translation of a subset of mRNAs localized in neurites through 3′ untranslated region (3′UTR)–dependent mechanisms (*14*–*17*). Recent studies have shown that a large population of mRNAs containing 3′UTR is distributed in dendrites through efficient mRNA transport (*18*–*20*). Some local translation repressors undergo rapid degradation in response to NMDAR activation, thereby up-regulating local protein synthesis in dendrites (*14*, *21*–*23*). Furthermore, while translation inhibitors alone diminish late-phase LTP and long-term depression induced by metabotropic glutamate receptor activation, coapplication of translation inhibitors and proteasome blockers largely restores them (*24*, *25*), suggesting that the tightly regulated balance between local protein degradation and synthesis underlies crucial memory processes and learning.

Kinesin superfamily proteins (KIFs) are microtubule-based motor proteins that transport various types of cargos to a specific area where they function, even to the distal neurites if necessary. This intracellular transport system is essential for neuronal morphogenesis, function, and survival (*26*). KIF17, a member of the kinesin-2 family, is abundantly expressed in neurons and has been suggested to transport NMDAR subunit 2B (NR2B)–containing vesicles in neuronal dendrites (*27*, *28*). Calcium-calmodulin-dependent protein kinase II (CaMKII)–dependent phosphorylation of KIF17 triggered by NMDAR-mediated activity regulates its capacity for binding/release of NR2B-containing vesicles, thereby underlying multiple phases of memory processes (*29*, *30*). Because the KIF17 has been shown to maintain synaptic NMDAR subunit 2A (NR2A)/NR2B levels in dendrites (*28*), we hypothesized that KIF17 might be functionally regulated by NMDAR-mediated neuronal activity.

## RESULTS

### KIF17 is degraded by the UPS in an activity-dependent manner

**Fig. 1:**
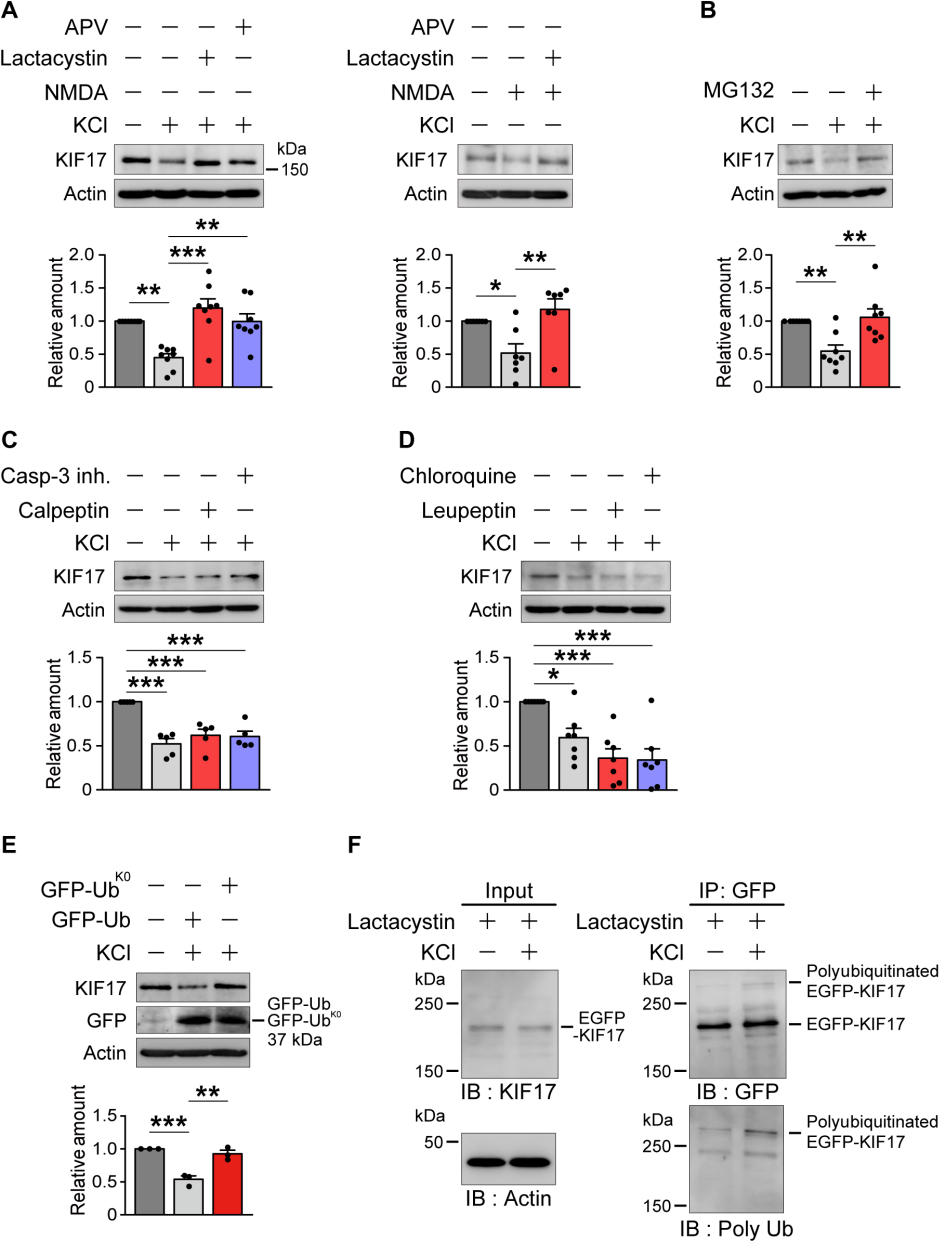
Activity-dependent proteasomal degradation of KIF17. (**A**) Immunoblotting of dissociated mouse hippocampal neurons (DIV 21 to 24) after stimulation with high KCl (60 mM; left) or NMDA (50 μM; right) for 5 min and with (+) or without (−) the indicated reagents. Data are expressed as the mean ± SEM throughout the figures unless otherwise mentioned. *P* < 0.01, one-way analysis of variance (ANOVA); *P < 0.05, **P < 0.01, ***P < 0.001, Bonferroni’s post hoc comparison. High-KCl stimulation, *n* = 8 independent experiments; NMDA stimulation, *n* = 7 independent experiments. See also fig. S2 (A and B). (**B**) MG132 prevented high KCl–induced degradation of KIF17. *P* < 0.01, one-way ANOVA; **P < 0.01, Bonferroni’s post hoc comparison. *n* = 8 independent experiments. See also fig. S2B. (**C**) Neither calpeptin nor caspase-3 inhibitor prevented the high KCl–induced decrease in KIF17. *P* < 0.001, one-way ANOVA; ***P < 0.001, Bonferroni’s post hoc comparison. *n* = 5 independent experiments. See also fig. S2B. (**D**) Neither leupeptin nor chloroquine prevented the high KCl–induced decrease in KIF17. *P* < 0.001, one-way ANOVA; *P < 0.05, ***P < 0.001, Bonferroni’s post hoc comparison. *n* = 7 independent experiments. See also fig. S2B. (**E**) Polyubiquitin-dependent degradation of KIF17. Hippocampal neurons (DIV 21 to 23) transfected with GFP-Ub or GFP-Ub^K0^ were stimulated with high KCl. *P* < 0.001, one-way ANOVA; **P < 0.01, ***P < 0.001, Bonferroni’s post hoc comparison. *n* = 3 independent experiments. (**F**) Ubiquitination of EGFP-KIF17. Hippocampal cultured neurons (DIV 21) expressing EGFP-KIF17 were pretreated with lactacystin A, stimulated with high KCl, and lysed. The samples of input and immunoprecipitated complexes including EGFP-KIF17 were analyzed by immunoblotting using antibodies specific for KIF17 and actin as well as GFP and polyubiquitin conjugates, respectively. IP, immunoprecipitation; IB, immunoblotting. See also fig. S2 (C and D).

We first imaged the KIF17 expression in dissociated mouse hippocampal culture neurons at 21 days in vitro (DIV) (fig. S1) and then compared the KIF17 levels in dissociated neurons (DIV 21 to 24) before and after depolarization with high-KCl treatment (60 mM, 5 min) (Fig. 1A). Endogenous KIF17 was rapidly degraded within 3 min after high KCl–induced stimulation (Fig. 1A), which did not affect any other related proteins including NR2B (fig. S2A). Pretreatment of neurons with the proteasome inhibitor lactacystin A (10 μM, 15 min) and the NMDAR antagonist d(-)-2-amino-5-phosphonovaleric acid (APV) (50 μM, 50 min) substantially prevented the high KCl–induced loss of KIF17 levels (Fig. 1A). Neuronal stimulation by NMDA alone (50 μM, 5 min) was sufficient to induce rapid degradation of KIF17 within 3 min after stimulation, and lactacystin A pretreatment inhibited this effect (Fig. 1A). Another kind of proteasome inhibitor, MG132, also substantially reversed the high KCl–induced degradation of KIF17 (Fig. 1B). In neurons, there are several kinds of modulators implicated in synaptic plasticity and memory that remove target proteins in an activity-dependent manner other than the proteasome (*9*). To investigate whether calpain, caspase-3, and lysosomes are implicated in KIF17 degradation, we individually applied calpeptin (10 μM, 20 min), caspase-3 inhibitor II (10 μM, 20 min), leupeptin (100 μg/ml, 60 min), and chloroquine (200 μM, 60 min) to dissociated hippocampal neurons before high KCl–induced stimulation. All of them failed to inhibit the degradation of KIF17 (Fig. 1, C and D). All pretreatments as above without stimulation did not produce notable changes in the KIF17 levels (fig. S2B). Collectively, these results suggested that the activity-regulated proteasomal degradation of KIF17 was induced by NMDAR-mediated activation.

To determine whether ubiquitination is required for KIF17 degradation, we transfected green fluorescent protein (GFP)–fused wild-type ubiquitin (GFP-Ub) and GFP-fused ubiquitin mutants with all seven lysines replaced by arginines (GFP-Ub^K0^) into hippocampal culture neurons. GFP-Ub is known to work on ubiquitin-dependent proteasomal degradation, whereas GFP-Ub^K0^ prevents it by blocking polyubiquitination of the target protein (*31*). KIF17 degradation after depolarization with high KCl was substantially blocked in neurons expressing GFP-Ub^K0^ but not GFP-Ub (Fig. 1E). To further analyze the ubiquitination of KIF17, enhanced GFP (EGFP)–tagged KIF17 (EGFP-KIF17) was transfected into hippocampal culture neurons (fig. S2C). Consistent with the results of endogenous KIF17 dynamics, we observed substantial degradation of ectopically expressed EGFP-KIF17 upon high KCl–induced stimulation (fig. S2D). Next, we stimulated the hippocampal neurons expressing EGFP-KIF17 after pretreatment with lactacystin A (10 μM, 15 min). The complex including EGFP-KIF17 was immunoprecipitated from neuronal lysates, and the ubiquitination of KIF17 was detected by antibodies against GFP and polyubiquitin conjugates (Fig. 1F) (*32*). High–molecular weight polyubiquitin labeling was observed, suggesting that the degradation of KIF17 depended on the activity-dependent ubiquitin pathway.

### KIF17 is locally degraded by the activity-induced UPS at dendrite

**Fig. 2:**
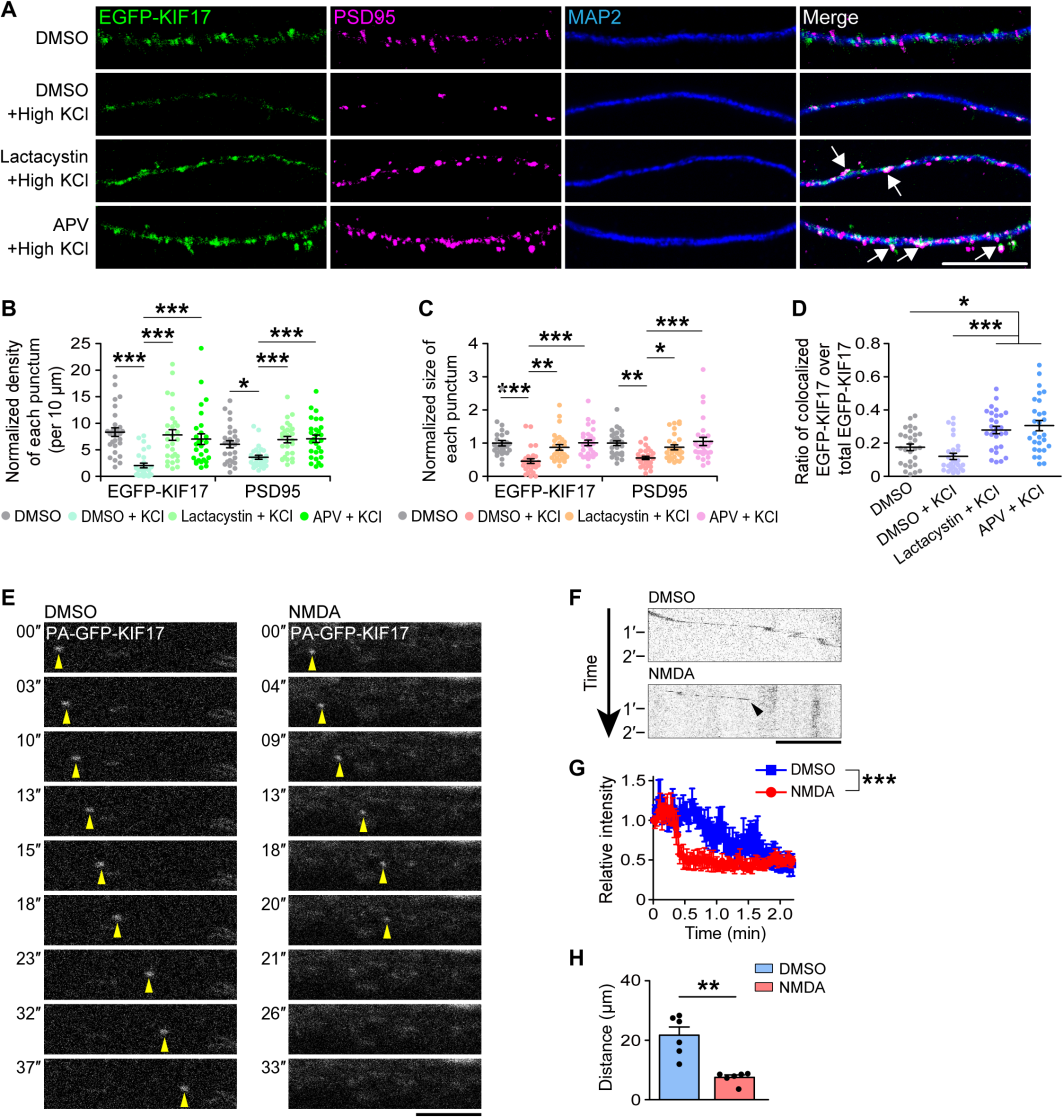
Neuronal stimulation triggers proteasome-mediated local degradation of KIF17. (**A**) Representative images of dendrites expressing EGFP-KIF17 that were immunostained with anti-PSD95 and anti-MAP2 antibodies. Scale bar, 10 μm. DMSO, dimethyl sulfoxide. (**B** and **C**) Quantification of the normalized density (B) and size (C) of the EGFP-KIF17 and anti-PSD95–positive puncta. *P* < 0.001, one-way ANOVA; *P < 0.05, **P < 0.01, ***P < 0.001, Bonferroni’s post hoc comparison. *n* = 28 dendrites from four independent cultures. (**D**) The ratio of the EGFP-KIF17 puncta colocalized with the anti-PSD95–positive puncta over the total EGFP-KIF17 puncta. *P* < 0.001, one-way ANOVA; *P < 0.05, ***P < 0.001, Bonferroni’s post hoc comparison. *n* = 28 dendrites from four independent cultures. See also figs. S3 and S4. (**E** and **F**) Time-lapse images of PA-GFP-KIF17 along dendrites (E) and their kymographs (F). Hippocampal neurons (DIV 7 to 8) transduced with a lentiviral construct containing PA-GFP-KIF17 were stimulated with NMDA (50 μM) after photoactivation of PA-GFP. Yellow arrowheads in (E), PA-GFP-KIF17. Black arrowhead in (F), the time and location of PA-GFP-KIF17 degradation. Scale bars, 5 μm. (**G**) Temporal variations in the relative fluorescence intensity under basal conditions (blue line) or upon NMDA stimulation (red line) for the PA-GFP-KIF17 puncta that were moving in dendrites. Effects of treatment (***P < 0.001) and time (*P* < 0.001), two-way ANOVA. *n* = 6 dendrites from six culture neurons. (**H**) Quantification of the distance that the PA-GFP-KIF17 puncta moved. **P < 0.01, two-tailed *t* test. *n* = 6 dendrites from six culture neurons.

To further analyze the NMDAR-mediated degradation of KIF17, we observed fixed hippocampal culture neurons (DIV 21) expressing EGFP-KIF17. EGFP-KIF17 puncta were localized throughout dendritic shafts, which was confirmed by colocalization with dendritic microtubule-associated protein 2 (MAP2) via immunocytochemical staining (fig. S3A). We also observed similar localizations for both the endogenous KIF17 and the ectopically expressed EGFP-KIF17 using antibodies against KIF17 and MAP2 (fig. S3B). To investigate the spatial location of KIF17 during its NMDAR-mediated degradation, we stimulated and fixed neurons expressing EGFP-KIF17. The density and size of the EGFP-KIF17 puncta along dendrites were reduced within 3 min after high KCl–induced stimulation (Fig. 2, A to C). Consistent with a previous report (*33*), the density and size of the immunostained postsynaptic density protein 95 (PSD95) puncta were also reduced. Pretreatment of neurons with lactacystin A (10 μM, 15 min) and APV (50 μM, 50 min) substantially inhibited the reduction in the density and size of the EGFP-KIF17 and the PSD95 puncta (Fig. 2, A to C). The ratio of EGFP-KIF17 colocalized with PSD95 over total EGFP-KIF17 was increased by lactacystin A or APV treatment (Fig. 2, A and D). Stimulation with NMDA alone (50 μM, 5 min) was sufficient to reduce the density and size of the EGFP-KIF17 and the PSD95 puncta. This effect was blocked by pretreatment with lactacystin A (10 μM, 15 min), and neurons exposed to lactacystin A showed increased colocalization between the EGFP-KIF17 and the PSD95 puncta (fig. S3, C to F). In addition, we further transfected with EGFP-KIF17 into hippocampal culture neurons expressing tag red fluorescent protein (tagRFP). Although the density and size of the EGFP-KIF17 puncta were not reduced upon high KCl–induced stimulation in somatic compartments, the EGFP-KIF17 degradation and its inhibition by lactacystin A and APV were reproduced in dendrites, suggesting that KIF17 was locally degraded (fig. S3, G to J). Individual treatments with lactacystin A and APV without stimulation and treatment with both lactacystin A and anisomycin (40 μM, 20 min) with stimulation all induced the EGFP-KIF17 accumulations at postsynaptic densities (fig. S4, A to D). These results indicated that EGFP-KIF17 regularly moved into postsynaptic densities even without stimulation, and the EGFP-KIF17 accumulations were not induced by local synthesis mechanisms.

Next, we imaged hippocampal culture neurons (DIV 7 to 8) expressing photoactivatable (PA)–GFP-tagged KIF17 (PA-GFP-KIF17) (*34*), following illumination with a 405-nm laser for photoactivation of PA-GFP. The velocity for anterogradely moving PA-GFP-KIF17 was 0.699 ± 0.05 μm/s, which is comparable to that of KIF17 movement described in previous reports (*28*, *35*). After NMDA application, PA-GFP-KIF17 stopped moving for 5.30 ± 1.39 s and disappeared at the dendrites (Fig. 2, E and F, and movie S1). The relative fluorescence intensities were rapidly decreased upon stimulation, and the distances that the PA-GFP-KIF17 puncta moved in the stimulated dendrites were shorter than those without stimulation because of the disappearance of the PA-GFP-KIF17 puncta (Fig. 2, G and H). These results suggested that NMDAR-mediated activity disrupted dendritic KIF17 transport. To further investigate the KIF17 dynamics in dendrites, in addition to PA-GFP-KIF17, we expressed PSD95-tagRFP in hippocampal neurons and then tested whether PA-GFP-KIF17 moved into the PSD95-tagRFP clusters. As a result, within stimulated and unstimulated dendrites, 5 of the 14 PA-GFP-KIF17 puncta moved into the PSD95-tagRFP clusters in total (fig. S4, E to G). In the NMDA-stimulated dendrites, although all seven PA-GFP-KIF17 puncta resulted in activity-dependent disappearance, 3 of the 7 PA-GFP-KIF17 puncta moved into the PSD95-tagRFP clusters (fig. S4, E to G). The velocities for the PA-GFP-KIF17 puncta moving into the PSD95-tagRFP clusters were significantly decreased compared with those for the PA-GFP-KIF17 puncta that did not move into the PSD95-tagRFP clusters (fig. S4H). To evaluate the interaction with the actin cytoskeleton, we applied cytochalasin D (1 μM, 30 min) to culture neurons expressing PA-GFP-KIF17 and PSD95-tagRFP and stimulated them. The velocity for PA-GFP-KIF17 puncta along the dendrites pretreated with cytochalasin D was 0.551 ± 0.06 μm/s, which turned out to be statistically comparable to the above velocity. All 6 PA-GFP-KIF17 puncta that disappeared in stimulated dendrites did not move into the PSD95-tagRFP clusters (fig. S4, E to G), suggesting that the KIF17 movement to postsynaptic densities depended on the actin cytoskeleton. Collectively, these data suggested that some proportion of KIF17 regularly moved into postsynaptic densities through actin cytoskeleton–based trafficking, and KIF17 was degraded within the dendrites, irrespective of localization. Thus, dendritic KIF17 transport was suggested to be dampened in an NMDAR-mediated activity–dependent manner.

### Local KIF17 synthesis and degradation of Kif17 mRNA occur along the dendritic shaft after activity-induced KIF17 degradation

**Fig. 3:**
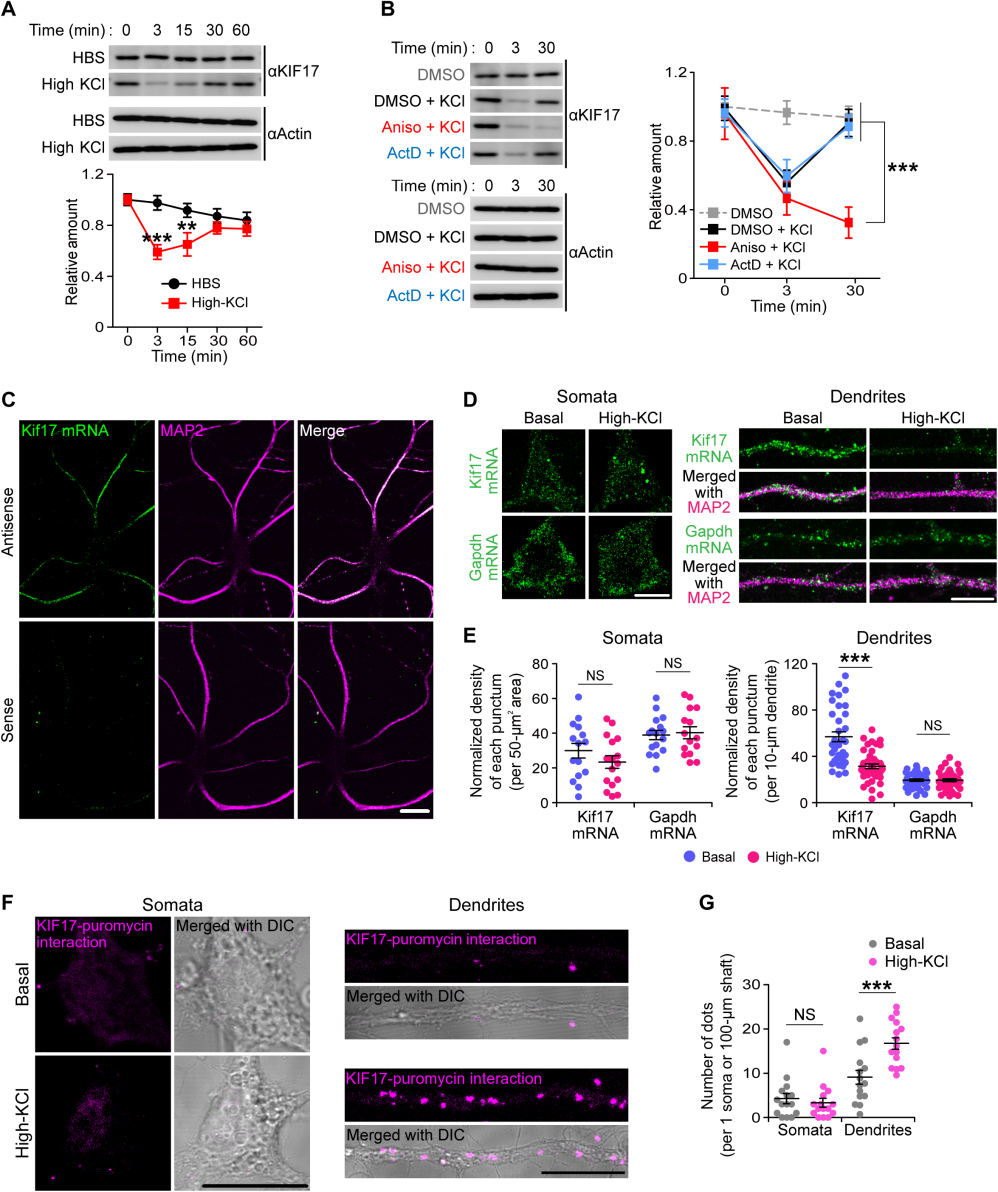
Local KIF17 synthesis and Kif17 mRNA degradation following the activity-induced KIF17 degradation. (**A**) Time courses of KIF17 expression after high KCl–induced stimulation. Effects of treatment (*P* < 0.01) and time (*P* < 0.001), two-way ANOVA; **P < 0.01, ***P < 0.001, Bonferroni’s post hoc comparison. *n* = 8 independent experiments. (**B**) Time courses of KIF17 expression in the stimulated hippocampal neurons treated with DMSO, anisomycin, or actinomycin D. Aniso, anisomycin; ActD, actinomycin D. Effects of treatment (*P* < 0.01) and time (*P* < 0.01) versus Aniso + KCl, two-way ANOVA; ***P < 0.001, Bonferroni’s post hoc comparison. *n* = 10 independent experiments. See also fig. S5A. (**C**) In situ hybridizations of hippocampal neurons (DIV 21) for Kif17 mRNA (green) combined with immunostaining for the dendritic shaft marker MAP2 (magenta). Scale bar, 20 μm. See also fig. S5C. (**D** and **E**) Representative images of quantitative in situ hybridization of hippocampal neurons (DIV 21 to 23) performed with the probes to detect Kif17 mRNA [top panel in (D)] and Gapdh mRNA [bottom panel in (D)] and their quantification of the normalized density of signals in somata [left panel in (E)] and dendrites [right panel in (E)]. A probe detecting Gapdh mRNA was used as a negative control. Scale bars, 10 μm. NS, *P* ≥ 0.05, ***P < 0.001, two-tailed *t* test. *n* = 15 to 16 somata and 34 to 44 dendrites from three independent cultures. See also fig. S5B. (**F** and **G**) Representative micrographs of the PLA between KIF17 and puromycin (F) and the quantification of the number of dots in somata and dendrites (G). The micrographs are merged with the differential interference contrast (DIC) images of DIV 21 dissociated hippocampal neurons. Scale bars, 20 μm. NS, *P* ≥ 0.05, ***P < 0.001, two-tailed *t* test. *n* = 15 somata and dendrites from three independent cultures.

Next, to further assess the molecular mechanisms in which KIF17 participates following synaptic activity, we focused on the regulation of KIF17 expression after its degradation. After the decrease in the levels of KIF17 in hippocampal culture neurons (DIV 21 to 24) was observed at 3 min from high KCl–induced stimulation as above, the KIF17 levels eventually returned to baseline until 30 min after stimulation (Fig. 3A). To test whether this recovery of expression depends on transcription and/or translation, we exposed neurons to anisomycin (40 μM, 20 min), a protein synthesis inhibitor, and actinomycin D (5 μM, 10 min), a transcription inhibitor, before stimulation with high KCl. Pretreatment with anisomycin completely blocked the recovery of KIF17 after degradation, whereas pretreatment with actinomycin D did not affect new synthesis of KIF17 (Fig. 3B). These pretreatments without stimulation did not cause any notable changes in the KIF17 levels (fig. S5A). Together, these data suggested that KIF17 was newly synthesized after degradation in a transcription-independent manner. In addition, we performed quantitative reverse-transcription polymerase chain reaction (qRT-PCR) to investigate the regulation of Kif17 mRNA when KIF17 was newly synthesized. As a result, the relative amount of Kif17 mRNA decreased at 30 min after high KCl–induced stimulation, and it tended to return to baseline until 60 min after stimulation (fig. S5B). The relative amount of Arc mRNA increased continuously, as was previously reported (*36*), while high KCl–induced stimulation did not produce a notable change in the Kif5c mRNA levels (fig. S5B). As some kinds of mRNAs localized at dendrites are involved in activity- and translation-dependent mRNA instability (*37*, *38*), these results led us to reason that Kif17 mRNA specifically down-regulated upon synaptic activity was localized and translated at dendrites.

Thus, we next examined whether Kif17 mRNA could be found in dendrites using fluorescence in situ hybridization (FISH) combined with immunostaining. We hybridized hippocampal neurons (DIV 21) with a digoxigenin-labeled antisense probe to Kif17 mRNA and immunostained them with anti-digoxigenin and anti-MAP2 antibodies. As a result, digoxigenin-positive signals were observed along the MAP2-positive neurites but not in somatic compartments, which indicated that Kif17 mRNA was mainly localized at dendritic shafts (Fig. 3C). Its specificity was confirmed by the lack of positive signals in in situ hybridizations with a sense probe and with cultured neurons from the *kif17* knockout mice (Fig. 3C and fig. S5C). Considering the results of FISH and qRT-PCR (Fig. 3C and fig. S5B), we also examined whether neuronal activation caused a compartmentalized reduction of Kif17 mRNA levels using quantitative FISH probes to detect the amount of transcript levels. The density of the Kif17 mRNA-positive puncta in dendrites, but not in somatic compartments, was significantly reduced within 30 min after high KCl–induced stimulation (Fig. 3, D and E). On the other hand, that of Gapdh-positive puncta was not changed both in somatic compartments and dendrites (Fig. 3, D and E). These results suggested that Kif17 mRNA localized at dendritic shafts was degraded in an activity-dependent manner. Furthermore, to visualize new KIF17 protein synthesis in dendritic and somatic compartments of hippocampal culture neurons (DIV 21), the puromycylation–proximity ligation assay (Puro-PLA) method (*39*) was conducted. We stimulated and fixed neurons with a brief period (15 min) of metabolic labeling and performed PLA between KIF17 and puromycin. The number of the signals representing the KIF17 synthesis was significantly increased in dendrites 20 min after high KCl–induced stimulation, compared with unstimulated dendrites, while there were few signals in somatic compartments regardless of the stimulation (Fig. 3, F and G). Together, these results suggested that neuronal activation induced local KIF17 synthesis and the compartmentalized degradation of Kif17 mRNA in dendrites.

### Local KIF17 synthesis at the dendritic shaft is driven by KIF17 3′UTR and poly(A)-binding protein–interacting protein 2A degradation

**Fig. 4:**
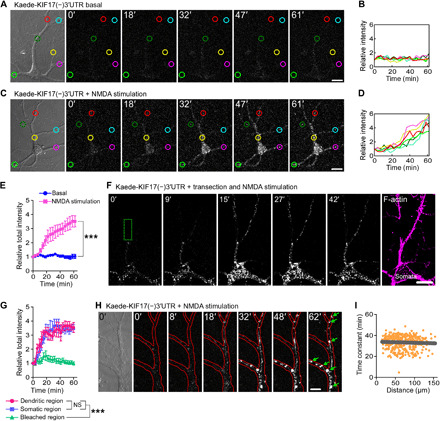
KIF17 3′UTR–mediated localized synthesis of Kaede occurs at the dendritic shaft. (**A** to **E**) Time-lapse images demonstrating the activity-induced synthesis of Kaede driven by KIF17 3′UTR upon NMDA stimulation (A and C) and their temporal variations in the individual (B and D) and total (E) relative intensity of Kaede. Each of the six measured areas in (A) and (C) was randomly chosen, and the circles’ colors of the measured areas correspond to the line colors in (B) and (D). Scale bars, 20 μm. Effects of treatment (***P < 0.001) and time (*P* < 0.001), two-way ANOVA. *n* = 6 neurons from five independent cultures. See also figs. S6 and S7 (A to I). (**F** and **G**) Time-lapse images demonstrating the local synthesis of Kaede upon NMDA stimulation in isolated dendrites (F) and their temporal variations in the total relative intensity of Kaede (G). Kaede proteins synthesized in isolated dendrites did not diffuse into the bleached region (the dotted green rectangle) because of their aggregations. The neurons were fixed and immunostained with an anti–F-actin antibody after the live observations, confirming isolation of the dendrites (the most right panel). Scale bar, 20 μm. Effects of treatment (***P < 0.001; NS, *P* ≥ 0.05) and time (*P* < 0.001), two-way ANOVA. *n* = 6 neurons from five independent cultures. (**H**) High-magnification observation of the local synthesis of Kaede driven by KIF17 3′UTR. Red lines trace the morphology of dendrites. Scale bar, 10 μm. (**I**) Median synthesis time constant plotted against the punctum distance from a single arbitrary point (gray line). Time constants of the individual puncta are shown as orange dots. A total of 348 Kaede puncta that were synthesized in response to NMDA-, high KCl–, and glutamate-induced activations were plotted.

KIF17 appeared to be locally synthesized in an activity-dependent but not transcription-dependent pathway. Therefore, it is important to determine the mechanism and precise location of KIF17 synthesis induced by neuronal activation. First, for this purpose, we transfected the photoconvertible translation reporter Kaede (*40*) into hippocampal culture neurons (DIV 21) that was uniformly expressed and photoconverted from green to red by illumination with a 405-nm laser (fig. S6A). When the neurons were stimulated with NMDA (50 μM, 5 min), temporal variations in the relative fluorescence intensity of the original (green) and photoconverted (red) Kaede did not change throughout the observations (fig. S6, B to D). Next, to investigate whether activity-dependent KIF17 synthesis is mediated by 3′UTR-dependent mechanisms, we transfected the Kaede fused to KIF17 3′UTR [Kaede-KIF17(−)3′UTR; (−) indicates that KIF17 coding region is not included] into hippocampal neurons (DIV 21 to 24) and then tested whether the neuronal activation induced local protein synthesis by recording the temporal intensities of green Kaede fluorescence after photoconversion. Although Kaede proteins were slightly expressed in contrast to transfection with Kaede only, high KCl– (60 mM, 5 min), glutamate- (25 μM, 5 min), and NMDA-induced (50 μM, 5 min) activation caused the appearance of green Kaede fluorescence (Fig. 4, A to D; figs. S6, E to I, and S7, A to F; and movie S2) in whole neurons. The temporal variations in the total relative fluorescence intensity upon stimulation induced by each reagent were significantly different from those without stimulation (Fig. 4E and fig. S7I). We additionally tested whether NMDA stimulation induced activity-dependent protein synthesis in the hippocampal neurons transfected with the Kaede fused to control UTR derived from a certain region located downstream of KIF17 3′UTR. NMDA stimulation did not induce new protein synthesis (fig. S7, G to I), suggesting that KIF17 3′UTR is essential for the activity-induced Kaede synthesis.

To test whether local Kaede synthesis driven by KIF17 3′UTR occurred in dendrites, we isolated dendrites by laser transection at the proximal regions and then observed activity-induced Kaede synthesis upon NMDA stimulation. Kaede proteins were locally synthesized at distal dendritic regions even when the dendrites were isolated (Fig. 4, F and G, and movie S3), consistent with the results of Puro-PLA (Fig. 3, F and G), and Kaede proteins synthesized in isolated dendrites did not diffuse into the bleached region. Locally synthesized Kaede has been shown to not freely diffuse and tends to aggregate even at low concentrations because of Kaede’s tetrameric nature and its translation in a restricted space such as synapses and dendritic shafts (*14*, *40*). This feature probably contributed to an increase in the intensity of the Kaede puncta (Fig. 4E). High-magnification observation of the activity-dependent synthesis of Kaede driven by KIF17 3′UTR revealed that Kaede puncta were synthesized within the dendritic shafts and suspended there because of aggregate formation (Fig. 4H and movie S4). Furthermore, we analyzed whether local KIF17 synthesis was regulated in a spatially restricted way. We plotted the median synthesis time constant as a function of the distance between each punctum and a single arbitrary point in the cell body. We found that the median synthesis time constant was uniform regardless of the punctum location (Fig. 4I). Collectively, these findings suggested that KIF17 was locally translated at dendritic shafts in an activity-dependent and 3′UTR-regulated manner.

**Fig. 5:**
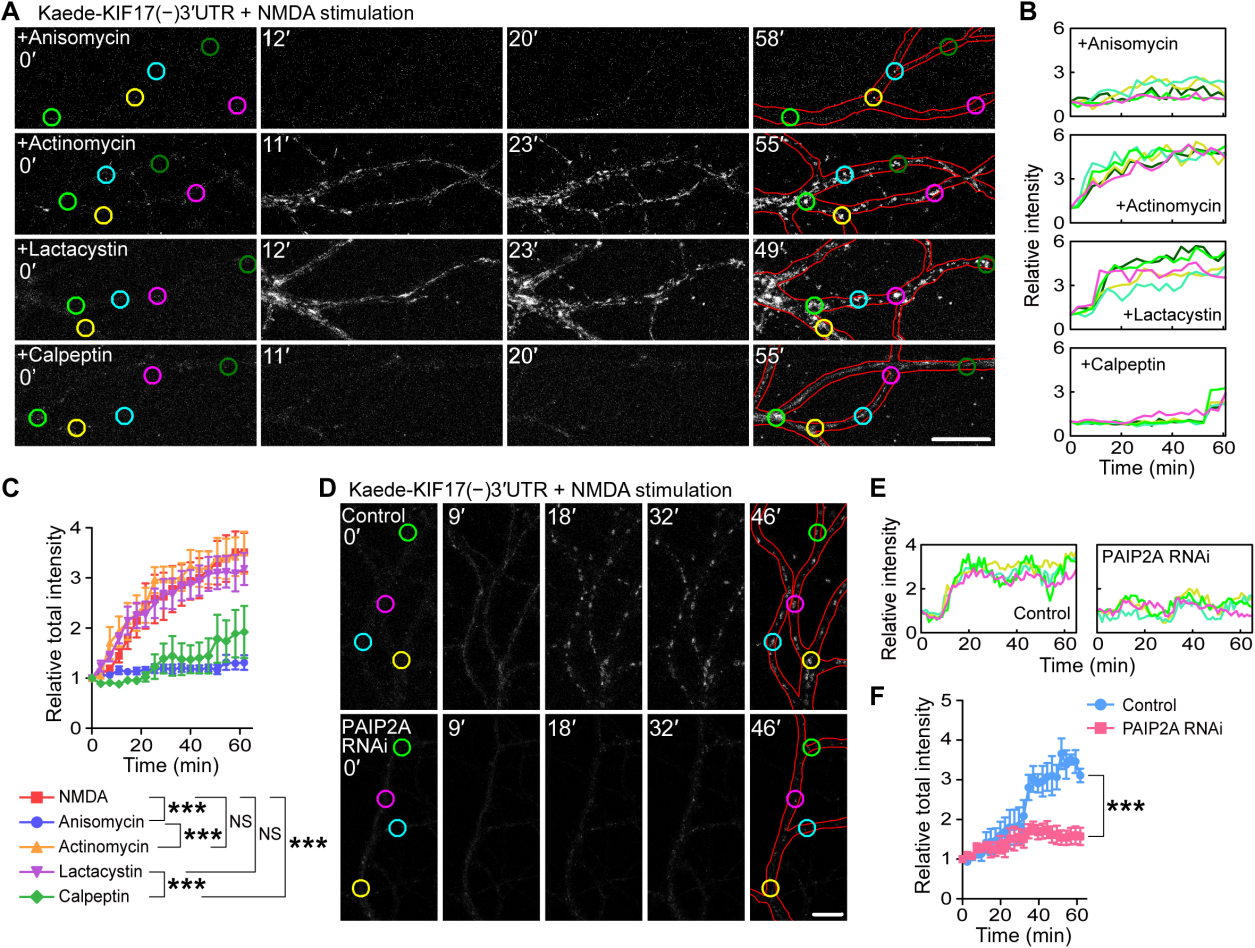
KIF17 3′UTR–mediated localized synthesis is driven by PAIP2A degradation. (**A** to **C**) Time-lapse images demonstrating the KIF17 3′UTR–mediated local synthesis of Kaede upon NMDA stimulation (A) and their temporal variations in the individual (B) and total (C) relative fluorescence intensity of Kaede. Neurons were pretreated with anisomycin (top), actinomycin D (second), lactacystin A (third), or calpeptin (bottom), before the stimulation. Each of the five measured areas in (A) was randomly chosen, and the circles’ colors of the measured areas correspond to the line colors in (B). Red lines trace the morphology of the dendrites. Scale bar, 20 μm. Effects of treatment (***P < 0.001; NS, *P* ≥ 0.05) and time (*P* < 0.001), two-way ANOVA. *n* = 5 dendrites from four independent cultures. (**D** to **F**) Time-lapse images demonstrating the PAIP2A involvement in local KIF17 synthesis (D) and their temporal variations in the individual (E) and total (F) relative fluorescence intensity of Kaede. Hippocampal culture neurons transfected with control shRNA (top) or shRNA against PAIP2A (bottom), in addition to Kaede-KIF17(−)3′UTR, were stimulated with NMDA. Each of the four measured areas in (D) was randomly chosen, and the circles’ colors of the measured areas correspond to the line colors in (E). Red lines trace the morphology of the dendrites. Scale bar, 10 μm. Effects of treatment (***P < 0.001) and time (*P* < 0.001), two-way ANOVA. *n* = 5 dendrites from five independent cultures. See also fig. S7 (J and K).

Pretreatment with anisomycin (40 μM, 20 min) completely blocked on-site reporter synthesis, while pretreatment with actinomycin D (5 μM, 10 min) did not affect new synthesis of the translation reporter (Fig. 5, A and B, and movie S5). This result suggested that local translation at the dendritic shaft was not dependent on the longer time scale of transcription. Furthermore, we observed that pretreatment of neurons with calpeptin (10 μM, 20 min), but not that with lactacystin A (10 μM, 20 min), attenuated local synthesis (Fig. 5, A and B, and movie S5). The temporal variations in the total relative fluorescence intensity revealed that pretreatment with anisomycin and calpeptin inhibited KIF17 3′UTR–driven new protein synthesis (Fig. 5C). In neurons, the poly(A)-binding protein (PABP)–interacting protein 2A (PAIP2A) is a negative translational regulator that is degraded by calpains in an activity-dependent manner (*23*). To determine whether degradation of PAIP2A by calpains enhanced the local translation of Kif17 mRNAs, we measured the NMDA-induced KIF17 3′UTR–mediated synthesis of Kaede after RNA interference (RNAi) knockdown of PAIP2A in hippocampal culture neurons. We observed that the RNAi-mediated loss of PAIP2A abrogated the NMDA-induced increase in Kaede fluorescence (Fig. 5, D to F, and movie S6) after confirming that RNAi knockdown of PAIP2A effectively worked and had no effects on KIF17 expression and Kif17 mRNA transcription levels (fig. S7, J and K). These data suggested that degradation of PAIP2A by calpains released translationally suppressed Kif17 mRNA, thereby enhancing KIF17 3′UTR–mediated local translation at the dendritic shaft.

### Newly synthesized KIF17 participates in microtubule-based transport

**Fig. 6:**
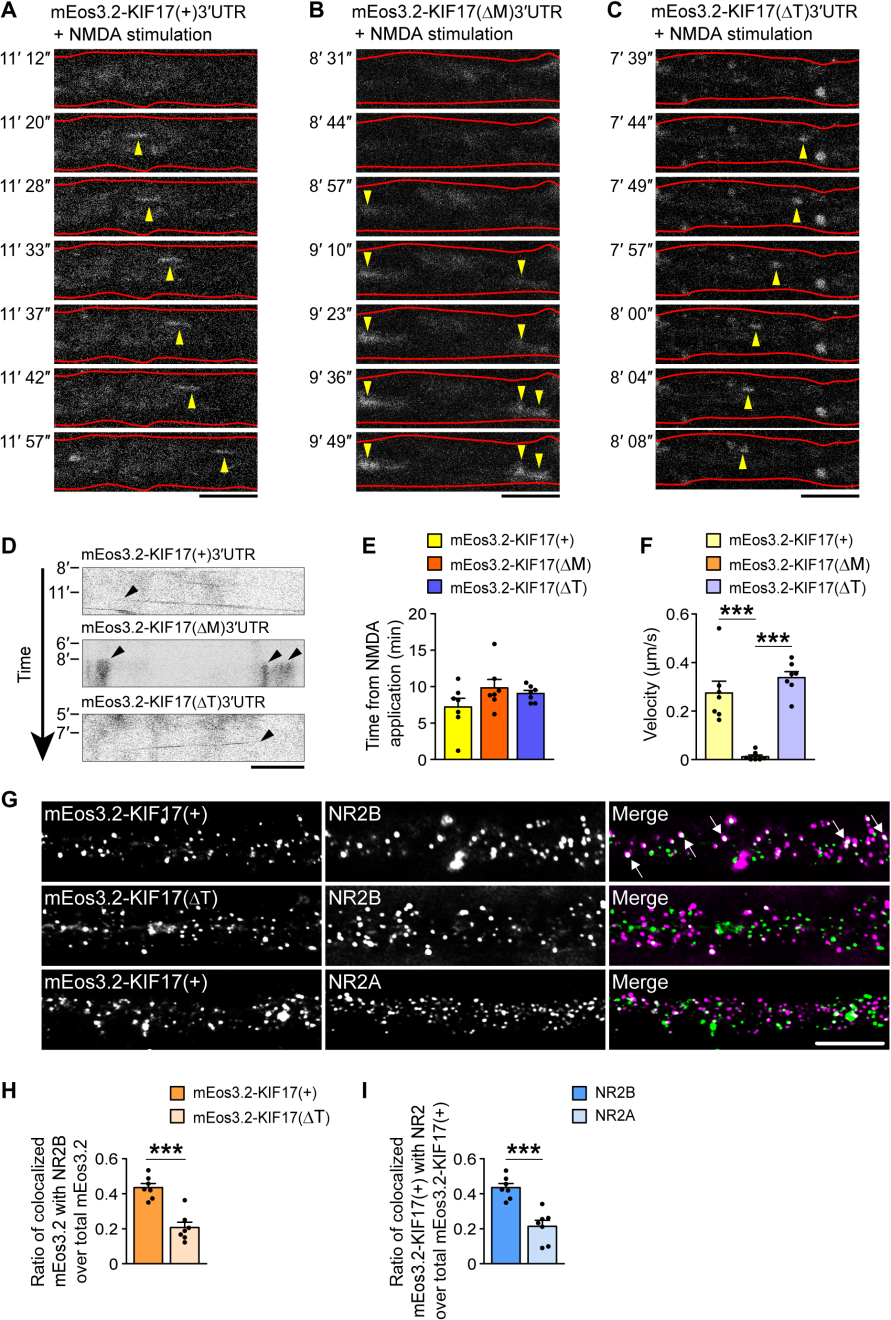
Newly synthesized mEos3.2-KIF17 moves along the dendrites and colocalizes with NR2B. (**A** to **D**) Time-lapse images of newly synthesized mEos3.2-KIF17(+) (A), mEos3.2-KIF17(ΔM) (B), and mEos3.2-KIF17(ΔT) (C) along dendrites and their kymographs (D). Hippocampal neurons (DIV 17 to 19) transfected with mEos3.2-KIF17(+)3′UTR, mEos3.2-KIF17(ΔM)3′UTR, and mEos3.2-KIF17(ΔT)3′UTR were stimulated with NMDA (20 μM) after photoconversion of mEos3.2. Yellow arrowheads in (A) to (C), newly synthesized mEos3.2 fused to each construct of KIF17. Black arrowheads in (D), the time and location of KIF17 3′UTR–mediated local synthesis. Red lines in (A) to (C) trace the morphology of the dendrites. Scale bars, 5 μm. See also fig. S8. (**E** and **F**) Quantifications as average time when mEos3.2 fused to each construct of KIF17 was synthesized from NMDA bath application (E) and velocities for movements of mEos3.2 fused to each construct of KIF17 (F). *P* ≥ 0.05 (E), *P* < 0.001 (F), one-way ANOVA; ***P < 0.001, Bonferroni’s post hoc comparison (F). *n* = 7 moving puncta from six culture neurons. (**G** to **I**) Representative images of dendrites expressing mEos3.2-KIF17(+) or mEos3.2-KIF17(ΔT), which were immunostained with anti-NR2B or anti-NR2A antibodies (G), and comparisons of the colocalization of the anti-NR2B–positive puncta (H) and that of mEos3.2-KIF17(+) (I) in dendrites. Scale bar, 5 μm. ***P < 0.001, two-tailed *t* test. *n* = 7 dendrites from seven culture neurons.

We next sought to determine whether newly synthesized KIF17 got mobility to participate in microtubule-based transport of its cargo. Since KIF17 coding region alone was insufficient for the induction of local synthesis at dendritic shafts (fig. S8, A and B), we expressed full KIF17 coding region with its 3′UTR in hippocampal culture neurons (DIV 17 to 19) and used the photoconvertible translation reporter mEos3.2 (*41*) as a fusion protein [mEos3.2-KIF17(+)3′UTR; (+) indicates that KIF17 coding region is included]. mEos3.2 circumvents aggregate formation because of its monomeric nature, in contrast to Kaede (*41*). Consistent with the analyses of local KIF17 synthesis, we recorded the motility of the newly synthesized green mEos3.2-KIF17(+) after photoconverting mEos3.2 from green to red. Following NMDA application (20 μM), the newly synthesized mEos3.2-KIF17(+) puncta appeared at dendritic shafts and then moved along the dendrites, while mEos3.2-KIF17(+) puncta were not synthesized without stimulation or with stimulation in the presence of anisomycin (Fig. 6A; fig. S8, C to E; and movie S7). Most mEos3.2-KIF17(+) puncta moved in an anterograde manner (five of seven moving puncta; Fig. 6, A and D, and movie S7). To confirm the relevance of the motility of the newly synthesized mEos3.2-KIF17(+), we expressed mEos3.2-KIF17(ΔM)3′UTR and mEos3.2-KIF17(ΔT)3′UTR, which lack the motor and tail regions of KIF17, respectively. Although the average time when they appeared at dendritic shafts from NMDA application was similar, the velocity for movement of mEos3.2-KIF17(ΔM) was substantially decreased compared with that of mEos3.2-KIF17(+) and mEos3.2-KIF17(ΔT) (Fig. 6, A to F, and movie S7). Moreover, to investigate the cargo transported by the newly synthesized KIF17, we fixed and immunostained the NMDA-stimulated neurons expressing the mEos3.2-KIF17(+)3′UTR or mEos3.2-KIF17(ΔT)3′UTR using anti-NR2 antibodies. The ratio of mEos3.2-KIF17(+) colocalized with NR2B was higher than that of mEos3.2-KIF17(ΔT), and the ratio of NR2B colocalized with mEos3.2-KIF17(+) was also higher than that of NR2A (Fig. 6, G to I).

### KIF17 synthesis driven by its 3′UTR is essential for fear extinction

**Fig. 7:**
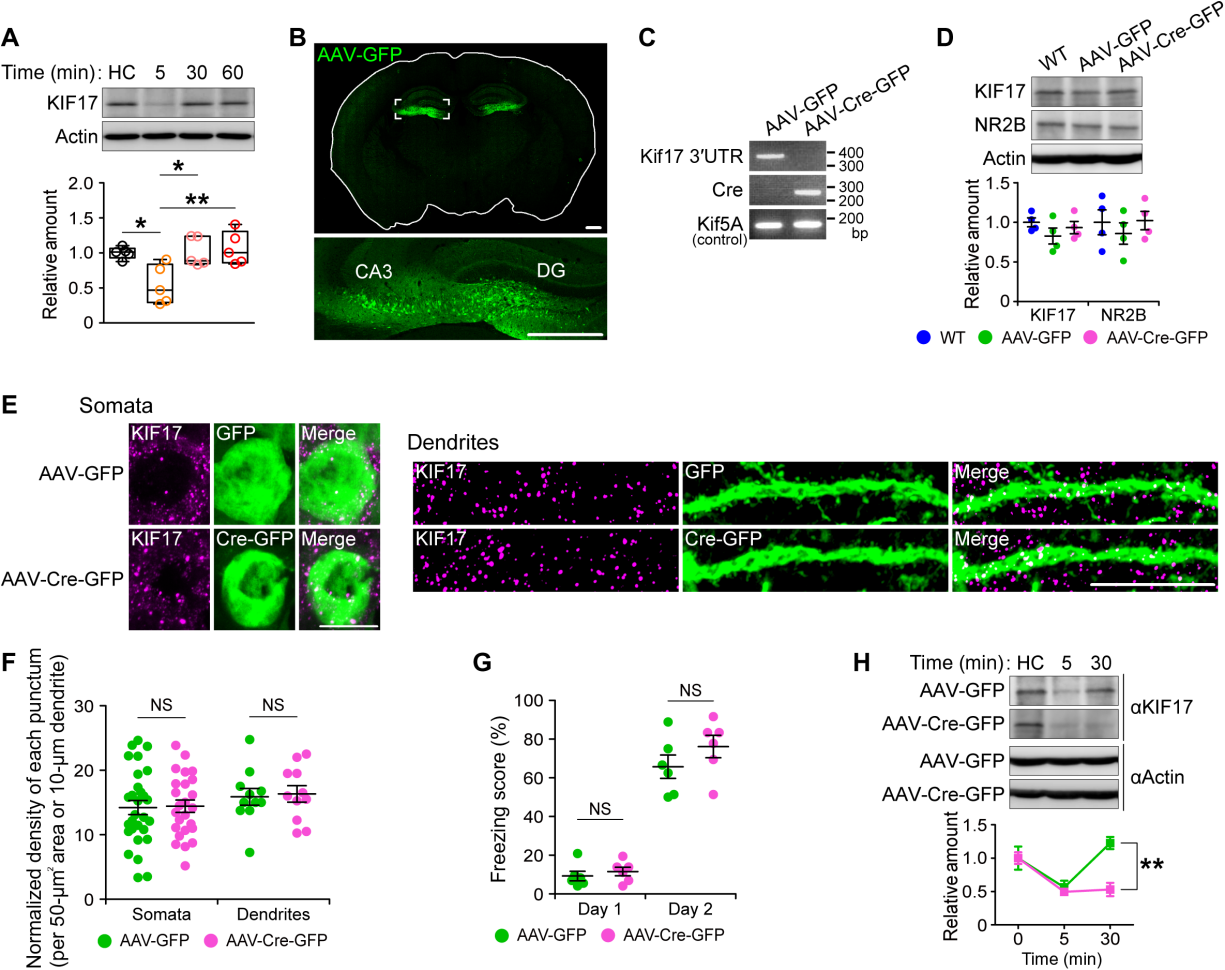
KIF17 3′UTR is essential for KIF17 synthesis induced by fear memory retrieval. (**A**) Time course of KIF17 expression in the mouse hippocampus after contextual fear memory retrieval. HC, home cage mice. In box plots, the central line represents the median, the edges of the box represent the interquartile range, and whiskers represent the minimum to the maximum. *P* < 0.01, one-way ANOVA; *P < 0.05, **P < 0.01, Bonferroni’s post hoc comparison. *n* = 5 mouse groups. See also fig. S9. (**B**) Representative images of a coronal brain section from the mouse bilaterally microinjected AAV-GFP into the hippocampi. Tile scanning with an LSM780 confocal laser scanning microscope was used. The box in the top panel represents the magnified region in the bottom panel. DG, dentate gyrus. Scale bars, 500 μm. See also fig. S10 (A to D). (**C**) Genomic PCR of the indicated hippocampal lysates. bp, base pair. (**D**) Comparison of the endogenous KIF17 levels in the hippocampus. *P* ≥ 0.05, one-way ANOVA. *n* = 4 mouse groups. WT, wild-type. (**E** and **F**) Representative images of immunofluorescence histochemistry demonstrating KIF17 expression in AAV-infected neurons (left, somata; right, dendrites) at the CA3 region of coronal brain sections (E) and quantification of the normalized density of anti-KIF17–positive puncta in somata and dendrites (F). Scale bars, 10 μm. NS, *P* ≥ 0.05, two-tailed *t* test. *n* = 25 to 29 somata and 11 dendrites from three mouse pairs. See also fig. S10 (E to H). (**G**) Freezing scores of the contextual fear conditioning test. NS, *P* ≥ 0.05, two-tailed *t* test. *n* = 6 mouse pairs. (**H**) Immunoblotting demonstrating the specific disruption of the synthesis of KIF17 by AAV-Cre–mediated deletion of *Kif17* 3′UTR. Effects of treatment (*P* < 0.05) and time (*P* < 0.01), two-way ANOVA; **P < 0.01, Bonferroni’s post hoc comparison. *n* = 3 mouse groups.

To address whether KIF17 degradation and subsequent synthesis are regulated by neuronal activity in vivo, we performed a contextual fear conditioning test. Eight- to 10-week-old male mice were fear-conditioned with an electric foot shock (FS) and exposed to the same context without the FS 24 hours later. The freezing score during memory retrieval (day 1) was higher than that during memory acquisition (day 2; fig. S9A). The hippocampi of the fear-conditioned mice were dissected and homogenized, and the expression levels of KIF17 were measured by immunoblotting. The levels of KIF17 were down-regulated within 3 min after completion of the memory retrieval protocol and subsequently returned to baseline until 30 min after that (Fig. 7A). On the other hand, the expression levels of KIF17 in the medial prefrontal cortex (mPFC) and the levels of KIF5C in the hippocampus were unchanged (fig. S9, B and C). These results suggested that the degradation and subsequent synthesis of KIF17 were induced by contextual fear memory retrieval in hippocampi because contextual fear memory is dependent on the activity of hippocampal neurons (*42*, *43*). This alteration of KIF17 expression was essentially reproduced in a cued fear conditioning test that was conditioned with a sound cue coupled with an FS (fig. S9, D to I).

To evaluate the hippocampal relevance of activity-induced KIF17 synthesis in fear memory, we established *Kif17* 3′UTR-flox (*Kif17* 3′UTR^flox/flox^) mice using the clustered regularly interspaced short palindromic repeat (CRISPR)–CRISPR-associated protein 9 (Cas9) system (*44*, *45*), after confirming the moderate amount of Kif17 mRNA expression in the mouse hippocampus (fig. S10, A to C). We bilaterally injected either Cre-GFP–expressing adeno-associated virus serotype 2 (AAV-Cre-GFP) or GFP only–expressing AAV2 (AAV-GFP) vectors into the hippocampi of the *Kif17* 3′UTR^flox/flox^ mice (*Kif17* 3′UTR^flox/flox^: Cre-GFP and *Kif17* 3′UTR^flox/flox^: GFP, respectively), as only *Kif17* coding region was insufficient for the induction of local translation in dendrites in vitro (fig. S8, A and B). Hippocampus-specific disruption of the *Kif17* 3′UTR was verified by fluorescence microscopy of the brain coronal section and genomic PCR of hippocampal lysates (Fig. 7, B and C, and fig. S10D). Moreover, to investigate whether KIF17 3′UTR influences the expression levels of KIF17 under basal conditions, we dissected and homogenized the hippocampi from the wild-type, *Kif17* 3′UTR^flox/flox^: GFP, and *Kif17* 3′UTR^flox/flox^: Cre-GFP mice, followed by measurements of the KIF17 levels by immunoblotting. The expression levels of KIF17 in the hippocampi of these three lines were comparable (Fig. 7D). Furthermore, to compare the KIF17 expression levels in AAV-infected neurons, we conducted fluorescence immunohistochemistry on hippocampal slices from the AAV-injected mice. We observed no substantial changes in the density of KIF17 immunostaining puncta in GFP-positive neurons at the CA3 region (Fig. 7, E and F). These results suggested that disruption of *Kif17* 3′UTR had no substantial effects on the KIF17 protein expression. The disruption of *Kif17* 3′UTR did not also influence the expression levels of NR2B and phospho-CREB [cAMP (cyclic adenosine 3′,5′-monophosphate) response element–binding protein] in AAV-infected neurons (Fig. 7D and fig. S10, E to H).

**Fig. 8:**
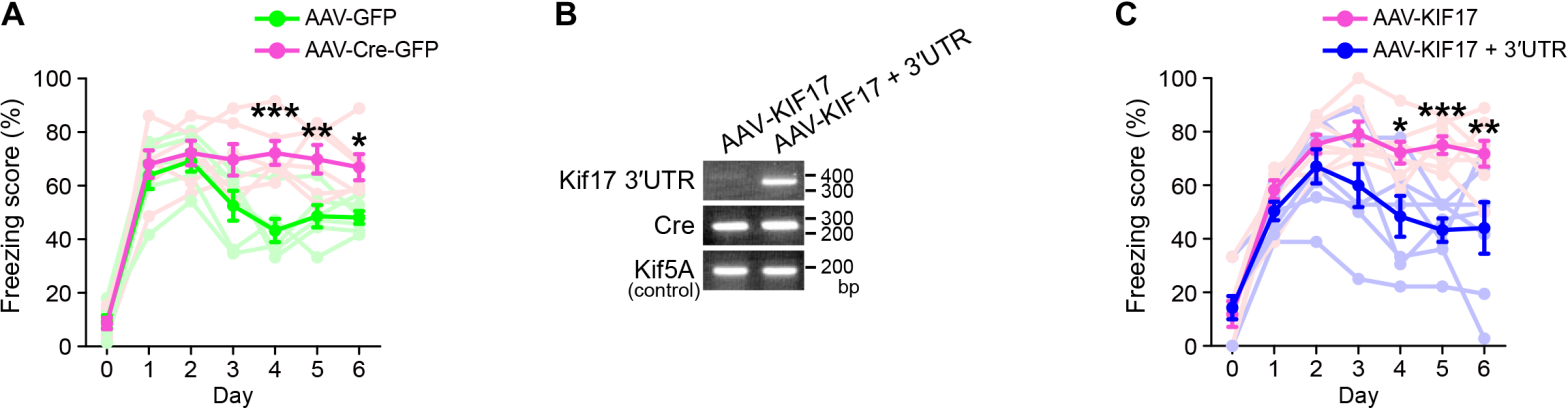
KIF17 3′UTR is essential for fear extinction. (**A**) Freezing scores during the contextual fear extinction test. The *Kif17* 3′UTR^2lox/2lox^ mice that received an AAV-Cre–mediated deletion of *Kif17* 3′UTR failed in fear extinction. Effects of treatment (*P* < 0.001) and time (*P* < 0.001), two-way ANOVA; *P < 0.05, **P < 0.01, ***P < 0.001, Bonferroni’s post hoc comparison. *n* = 6 mouse pairs. (**B**) Genomic PCR of the hippocampal lysates of the *Kif17* 3′UTR^2lox/2lox^ mice at 4 weeks after the injections of the indicated AAV vector including AAV-Cre-GFP. (**C**) Freezing scores during the contextual fear extinction test. An AAV-mediated *Kif17* 3′UTR introduction to the mice that received an AAV-Cre–mediated deletion of *Kif17* 3′UTR specifically rescued the fear extinction impairment but not *Kif17* without 3′UTR introduction. Effects of treatment (*P* < 0.001) and time (*P* < 0.001), two-way ANOVA; *P < 0.05, **P < 0.01, ***P < 0.001, Bonferroni’s post hoc comparison. *n* = 7 mouse pairs.

In a contextual fear conditioning test, the *Kif17* 3′UTR^flox/flox^: Cre-GFP mice exhibited similar freezing scores as the *Kif17* 3′UTR^flox/flox^: GFP mice during memory acquisition and retrieval (Fig. 7G). However, although fear memory retrieval induced both the degradation and subsequent synthesis of KIF17 in the hippocampi of the *Kif17* 3′UTR^flox/flox^: GFP mice, it induced only the degradation but not the subsequent synthesis of KIF17 in the hippocampi of the *Kif17* 3′UTR^flox/flox^: Cre-GFP mice (Fig. 7H). Therefore, it was suggested that *Kif17* 3′UTR was indispensable to the up-regulation of KIF17 expression following the fear memory retrieval–induced KIF17 degradation. Last, we asked whether disruption of *Kif17* 3′UTR could influence the extinction of contextual fear memory. We conditioned mice on day 0 with an FS and exposed them to the same context without an FS on six consecutive days. The freezing scores of the *Kif17* 3′UTR^flox/flox^: Cre-GFP mice and those of the *Kif17* 3′UTR^flox/flox^: GFP mice similarly increased until day 2, but only those of the *Kif17* 3′UTR^flox/flox^: Cre-GFP mice did not decrease on days 3 to 6 (Fig. 8A), suggesting a predominant role of *Kif17* 3′UTR in the extinction of contextual fear memory but not in the background fear level or in fear acquisition (Figs. 7G and 8A). We also examined whether ectopic expression of *Kif17* containing its 3′UTR in the hippocampus could rescue contextual fear extinction deficits in the *Kif17* 3′UTR^flox/flox^: Cre-GFP mice. Injection of *Kif17* containing its 3′UTR-expressing AAV serotype 9 (AAV-KIF17 + 3′UTR) (Fig. 8B) notably restored the reduction curve in freezing scores during the extinction trials in the *Kif17* 3′UTR^flox/flox^: Cre-GFP mice but that of only *Kif17-*expressing AAV9 (AAV-KIF17) failed in the restoration (Fig. 8C). These results collectively demonstrated that KIF17 synthesis driven by its 3′UTR was essential for extinction of fear memory.

## DISCUSSION

### NMDAR-mediated activity temporarily disrupts dendritic KIF17 transport

The experiments presented here demonstrate that KIF17 is rapidly degraded by the proteasome in response to NMDAR-mediated activity. Consistently, our analyses also suggest that dendritic KIF17 transport is temporarily disrupted along the dendritic shaft (Figs. 1 and 2). Ectopically expressed EGFP-KIF17 has been shown to transport NR2B-containing vesicles in dendrites (*30*). Therefore, the activity-dependent degradation of KIF17 reported here suggests that NMDAR-mediated activity dampens dendritic NR2B transport.

To date, it has been reported that KIF17 binds to NR2B through the Mint1-containing scaffolding protein complex (*27*, *35*). NMDAR-mediated activity triggers the disruption of the KIF17-Mint1 association through CaMKII-dependent phosphorylation of KIF17 on Ser^1029^ and results in unloading of NR2B from KIF17 in the vicinity of synapses (*29*, *30*). Thus, dendritic NR2B transport by KIF17 is terminated via the unloading mechanism triggered by neuronal activity, consistent with our present findings. Furthermore, the disruption of its capacity for NR2B binding/release leads to spatial memory impairment in vivo (*30*). It will be interesting to determine whether the activity-dependent phosphorylation of KIF17 is implicated in the activity-dependent degradation of KIF17.

Our present data demonstrate that some proportion of KIF17 moved into the postsynaptic densities regardless of the existence of neuronal stimulation. The results suggest that dendritic NR2B transport by KIF17 contributes to both general supply and activity-induced on-time delivery. Since the velocity for KIF17 moving into the postsynaptic density is slower than that for KIF17 moving along the dendrite (fig. S4H), the mechanism of KIF17 trafficking to the postsynaptic density might not be through adenosine triphosphate–driven microtubule-mediated active transport. The previous studies have shown that CaMKII mediates the activity-dependent recruitment of proteasome from dendritic shaft to synaptic spine (*46*, *47*). Further investigation is required to identify the shaft-to-spine trafficking mechanism of KIF17, such as the association with the actin-based cytoskeleton (*46*, *48*).

### NMDAR-mediated activity induces dendritic transport by newly synthesized KIF17, which is essential for cognitive flexibility

**Fig. 9:**
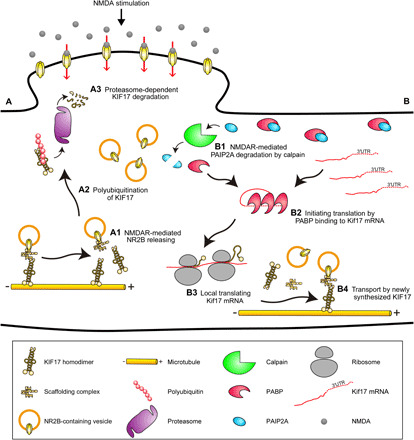
Models for activity-dependent local transport regulatory mechanisms via local degradation and synthesis of KIF17, leading to regulation of NR2B deposition. Models for the molecular mechanisms of local KIF17 degradation and synthesis. (**A**) Model for the molecular mechanism of local KIF17 degradation. (A1) KIF17 unloads NR2B in response to Ca^2+^ influx through NMDAR activation. (A2) KIF17 is polyubiquitinated in an activity-dependent manner. (A3) The proteasome proteolyzes polyubiquitinated KIF17. (**B**) Model for the molecular mechanism of local KIF17 synthesis. (B1) PAIP2A is degraded by calpain in the NMDAR-mediated pathway, leading to augmented binding of poly(A)-binding protein (PABP) to Kif17 mRNA. (B2) PABP binding to Kif17 mRNA facilitates translation initiation. (B3) Kif17 mRNA is locally translated at the dendritic shaft. (B4) Newly synthesized KIF17 participates in cargo transport along microtubules, suggesting the regulation of local deposition of NR2B along a dendritic shaft that is crucial for cognitive flexibility.

In this study, we demonstrated that NMDAR-mediated activity triggered local KIF17 synthesis driven by its 3′UTR (Fig. 4). The hypothesis that KIF17 is locally synthesized is supported by molecular biological analyses showing transcription-independent KIF17 up-regulation (Fig. 3B), local synthesis of endogenous KIF17 (Fig. 3, F and G), KIF17 3′UTR–driven Kaede synthesis (Fig. 4), and Kif17 mRNA localization in the dendritic shaft (Fig. 3C). We also demonstrated that activity-dependent local KIF17 synthesis was attenuated by calpeptin treatment and RNAi knockdown of PAIP2A (Fig. 5). PAIP2A is proteolyzed by calpains in an NMDAR-mediated activity-dependent manner, facilitating activation of mRNA translation (*23*). Initiating translation is enhanced by PABP because of the formation of a circular initiation complex through PABP binding to the poly(A) tail (*49*). Competition of PAIP2A with the poly(A) tail for binding to PABP reduces PABP–poly(A) tail interactions, thus inhibiting the initiation of translation (*50*). Together, our results suggest that the degradation of PAIP2A promotes the interaction between PABP and the poly(A) tail of Kif17 mRNA, resulting in localized translation of Kif17 mRNA (Fig. 9). Moreover, as the treatment with actinomycin D or lactacystin A also affected the trajectory of the increase in relative total Kaede intensity after NMDA stimulation (Fig. 5C), although this was not statistically significant, another mechanism other than the above might be involved in the activity-dependent local KIF17 synthesis.

The observation of motility and colocalization with NR2B of newly synthesized KIF17 in the NMDA-stimulated dendrites (Fig. 6) suggests that NMDAR-mediated neuronal activity triggered dendritic NR2B transport by locally synthesized KIF17. In this case, the product of the translation hotspot, KIF17, could move along a dendrite via microtubule-mediated active transport, although diffusion has been considered to be basically responsible for dendritic protein distribution from activity-dependent local protein synthesis sites (*51*). It has been reported that single spine–restricted stimulation by two-photon uncaging enhances the spine morphological plasticity, which depends on local proteasomal degradation or local translation (*52*, *53*), and local dendritic stimulation facilitates accumulation of the locally synthesized PSD95 in stimulated synapse and dendritic shaft (*54*). Therefore, local synaptic activation of an individual spine might induce the local KIF17 degradation and synthesis underlying the local regulation of KIF17 transport. Furthermore, as previously reported, activity-dependent transport by UNC-116, the *Caenorhabditis elegans* homolog of vertebrate kinesin-1 heavy chain (KIF5), mediates the dendritic delivery and redistribution of synaptic glutamate receptors, leading to the utilization of a synaptic glutamate receptor at multiple synapses within a single dendrite (*55*). Thus, whether a KIF17 translational hotspot could affect synaptic plasticity at a synapse that resides within the same dendrite but far away from the hotspot would be a topic of future research.

The signals representing the existence of Kif17 mRNA and the occurrence of local endogenous KIF17 synthesis were localized at dendritic compartments (Fig. 3, C and F); nevertheless, KIF17 proteins were expressed in somatic compartments (fig. S1), and the deletion of *Kif17* 3′UTR did not influence the NR2B levels in dendrites (fig. S10, E and F). These results provide the possibility that local KIF17 degradation and synthesis are not the only mechanisms involved in the regulation of KIF17 expression. The previous study has shown that KIF17 transports NR2B from somatic compartment to distal dendrite (*30*). Thus, in parallel with local degradation and synthesis, KIF17 expression might be regulated by another molecular mechanism, such as activity- and CREB-mediated regulation of KIF17 levels (*28*, *30*). This can explain why RNAi knockdown of PAIP2A does not lead to an increase in KIF17 levels (fig. S7J). Together, KIF17 would transport NR2B both from somatic compartment to distal dendrite and from local to local for the purpose of the global distribution and the local redistribution, respectively.

As not only deficits in fear extinction (*56*) but also local translation (*57*) have been focal points for dysregulation in disease, it will be crucial to address how locally synthesized KIF17 is involved in the NR2B delivery to the spine or extrasynaptic site. The disruption and regeneration of local dendritic KIF17 transport could be reflected in the dendrite-restricted regulation of multiple synaptic weights. Thus, the present study will provide an emerging concept that activity-dependent localized kinesin degradation and synthesis mediate the kinesin-driven regulation of the local spatiotemporal deposition of cargo, thereby leading to the induction of cognitive flexibility. This concept, “dendritic plasticity,” could help further understanding how a single dendrite serves as the computational unit for the memory process.

## MATERIALS AND METHODS

### Mice

Mice were maintained in a specific pathogen–free environment with free access to food and water. A *Kif17*-knockout mouse line was established previously (*28*).

A *Kif17* 3′UTR-flox mouse line was established using the CRISPR-Cas9 system (*44*, *45*) by the Takahashi laboratory (University of Tsukuba). Our plan was to insert the loxP sequence in the site between exon 15 and the 3′UTR of the *Kif17* gene and the site located downstream of *Kif17* 3′UTR (fig. S10B). A px330 vector (#42230, Addgene) was used to express guide RNA for the targeted region and Cas9 protein in mouse zygotes. Appropriate CRISPR target sites were identified, and double-stranded DNAs of 20 base pairs (bp) derived from the target regions (*Kif17* 3′UTR and surrounding regions) were inserted into the px330 vector. The cleavage activity of the px330 vectors containing guide sequences was confirmed using the EGxxFP system (*58*). We designed the donor vector containing corresponding loxP sites with 1873- and 2334-bp sequences homologous to each side surrounding each single-guide RNA–mediated double-strand break. To facilitate the detection of the intended insertions, we engineered the donor vector to contain Asc I and Eco RV restriction sites in addition to loxP sequences (fig. S10B).

Next, two px330 vectors containing guide sequences and the donor vector containing loxP sequences were injected into 294 mouse zygotes by the Takahashi laboratory. Eighteen pups were generated, and genomic DNA from the pups born after microinjection was amplified by PCR and subjected to digestion with Asc I and Eco RV separately. As a result, we identified one mouse that carried loxP sites and an intact *Kif17* 3′UTR in one allele, although *Kif17* 3′UTR was completely deleted in the other allele. To determine whether the donor vector and the px330 vectors containing guide sequences were integrated into the chromosomes, we performed PCR with mouse genomic DNA and a primer pair for the detection of Cas9 and ampicillin resistance genes. PCR products of Cas9 and the ampicillin resistance gene were not observed in the floxed mice. *Kif17* 3′UTR-flox mice were genotyped by PCR using the following primers: Pr1, 5′-CTAGAGTCCCTTGACATCCCCTTC-3′; Pr2, 5′-TGGCAGGCAAGTCAGTAGGG- 3′. The reaction was conducted for 30 cycles at 94°C for 30 s, 59°C for 30 s, and 72°C for 30 s using AmpliTaq DNA polymerase (Thermo Fisher Scientific). The genotypes were identified as shown in fig. S10C.

### Intrahippocampal injection of AAV

Five- to 6-week-old *Kif17* 3′UTR*^2lox/2lox^* male mice received intrahippocampal microinjections of AAV under anesthesia with isoflurane as previously described (*48*). The infusion of AAV vectors was performed through a 33-gauge stainless cannula (Plastics One Inc.) at 2.0 mm posterior and ± 1.7 mm lateral from the bregma and 2.0 mm in depth from the cerebral parenchyma surface, targeting the dorsal hippocampi. After 5 min from cannula insertion, 1 μl of vector per hippocampus was delivered at 0.2 μl/min. The cannula was left in place for 5 min and then withdrawn from the brain. Three to 4 weeks after the vector infusion, the mice were subjected to the contextual fear conditioning and extinction test series. To confirm the expression of AAV vector, the mice were anesthetized and subjected to perfusion fixation with 4% paraformaldehyde (PFA) in 0.1 M K-phosphate buffer [19 mM KH_2_PO_4_ and 81 mM K_2_HPO_4_ (pH 7.4)] 2 weeks after the vector infusion. The fixed samples were sectioned with MicroSlicer Zero 1 (Dosaka EM) at 50 μm thick. The sections were observed on an LSM780 confocal laser scanning microscope (Zeiss).

### AAV production

AAV-KIF17 + 3′UTR and AAV-KIF17 constructs were generated by inserting KIF17 complementary DNA (cDNA) with/without KIF17 3′UTR into the Eco RI and Hind III sites of the pAAV-vector (a gift from the Ohki laboratory, The University of Tokyo). AAV production was conducted by ES-Mouse/Virus Engineering and Resource of International Research Center for Neurointelligence (IRCN-E/VER, The University of Tokyo). AAVs were produced by cotransfection into AAV293 cells (in AAV Helper-Free System, 240071, Agilent Technologies) with the pAAV-vector containing KIF17 + 3′UTR or KIF17 sequence, the pAAV-RC9 vector, and the pHelper vector (gifts from the Ohki laboratory). AAV-GFP and AAV-Cre-GFP were purchased from the Vector Core at the University of North Carolina (UNC Vector Core).

### Behavioral analyses

All animal experiments were conducted under the University of Tokyo’s restrictions regarding animal experimentation, and approval was received from the Institutional Animal Care and Use Committee of the University of Tokyo Graduate School of Medicine. Notification numbers of these experiments are 1722T124, 1722T126, M-P15-118, and M-P15-119. All mice used for behavioral analyses were handled for five consecutive days (1 min/day). After 3 days of handling, they were separately isolated and handled for two more days. The conditioning was carried out using a chamber (18 cm by 17 cm by 30 cm) with a shock generator/scrambler, a cycle timer, and a conditioned stimulus controller (SGS-003DX, CBX-CT, and CSC-001; Muromachi). It had gray walls and grid floors with identical-size stainless steel rods. All mouse behaviors in the chamber were monitored by a video camera, and the fraction of the time that the mice spent freezing was manually calculated as a freezing score. All experiments were performed in a randomized single-blind fashion, and 8- to 10-week-old male C57BL/6J mice were used.

#### Contextual fear conditioning test

The contextual fear conditioning test was performed as previously described (*28*). Memory acquisition protocol consisted of a 180-s baseline period and followed a presentation of an electric FS (2 s, 0.8 mA). After the FS, a 60-s period was given and then the mice were returned to their home cages. At 24 hours after the conditioning, the mice were put back into the same chamber and kept in the chamber for 3 min.

#### Cued fear conditioning test

The cued fear conditioning test was performed on the basis of a previously described protocol (*48*). Memory acquisition protocol consisted of a 120-s baseline period and followed presentations of three conditioned stimulus (CS)–unconditioned stimulus (US) pairings (CS: tone, 15 s, 2800 Hz, 75 dB; US: electric FS, 2 s, 0.8 mA) each separated by intertrial intervals of 120 s. After third FS, a 90-s period was given and then the mice were returned to their home cages. The US coterminated with the CS. At 24 hours after the conditioning, the mice were put back into the same chamber. Memory retrieval protocol consisted of the same as memory acquisition protocol except for cancelling the US.

#### Contextual fear extinction test

The contextual fear extinction test was performed as previously described (*48*). Memory acquisition protocol consisted of a 180-s baseline period, following a presentation of an electric FS (2 s, 0.6 mA). After the FS, a 60-s period was given and then the mice were returned to their home cages (day 0). Starting 24 hours after the memory acquisition, daily extinction trials with a 3-min reexposure to the conditioned context were performed for six consecutive days (days 1 to 6).

### Cells

Dissociated hippocampal neuron cultures were prepared as described previously (*28*). Briefly, hippocampi were removed from 16.5-days post-coitum (dpc) embryo mouse brains and dissociated with 0.25% trypsin. Hippocampal neurons were then cultured in minimal essential medium supplemented with 1 mM sodium pyruvate (Gibco), 0.6% glucose, GlutaMAX I (#35050, Gibco/Thermo Fisher Scientific), and 2% B27 supplement mixture (Thermo Fisher Scientific) or 2% MACS NeuroBrew-21 (Miltenyi Biotec) up to DIV 24. They were plated on glass slides previously coated with polyethylenimine and poly-l-lysine.

### Cloning of lentiviral constructs

EGFP and PA-GFP (*34*) cassettes containing Asc I sites were amplified by PCR from the pEGFP-C1 vector (Clontech Laboratories Inc.) and pPA-GFP-C1 vector (#11910, Addgene), respectively. Then, these sequences were cloned into the Mlu I and Spe I sites of the pWPXL lentiviral vector (#12257, Addgene). The KIF17-fused N-terminal EGFP or PA-GFP lentiviral constructs were generated by inserting KIF17 cDNA (*29*) into the Asc I and Spe I sites of the pWPXL lentiviral vector expressing EGFP or PA-GFP. PSD-95-pTagRFP, GFP-Ub, and GFP-Ub^K0^ (Ub^K0^ indicates that all seven lysine residues were mutated to arginines) (*31*) (#52671, #11928, and #11934, respectively, Addgene) were used to construct lentiviral vectors. PSD-95-pTagRFP, GFP-Ub, and GFP-Ub^K0^ cassettes were amplified by PCR and cloned into the Mlu I and Spe I sites of the pWPXL lentiviral vector. TagRFP cassette was amplified by PCR from the pTagRFP-C1 vector (Clontech Laboratories Inc.) and cloned into the Mlu I and Spe I sites of the pWPXL lentiviral vector.

For generation of the translation reporter Kaede-KIF17(−)3′UTR, mEos3.2-KIF17(+, ΔM, and ΔT)3′UTR, and Kaede-Control UTR vectors, Kaede (*40*) and mEos3.2 (*41*) cassettes containing Asc I sites were amplified by PCR from the pKaede-MC1 vector (#AM-V0012M, MBL International) and mEos3.2-C1 vector (#54550, Addgene), respectively. Then, they were cloned into the Mlu I and Spe I sites of the pWPXL lentiviral vector. The KIF17 3′UTR (561 bp) and control UTR (a certain region located downstream of KIF17 3′UTR, 561 bp) were amplified by PCR from mouse genomic DNA and cloned into the Eco RI and Spe I sites of the pWPXL lentiviral vector expressing Kaede. The KIF17 3′UTR was also cloned into the Asc I and Spe I sites of the pWPXL lentiviral vector expressing mEos3.2. mEos3.2-KIF17(+, ΔM, and ΔT)3′UTR was constructed by PCR amplification of KIF17(+) (1 to 1038), KIF17(ΔM) (344 to 1038), and KIF17(ΔT) (1 to 846) and cloning into the Eco RI and Asc I sites of the pWPXL lentiviral vector containing the mEos3.2 and KIF17 3′UTR cassettes.

For knockdown of PAIP2A, short hairpin RNA (shRNA) cassettes were produced by annealing two cDNA oligonucleotides. The target sequences directed against PAIP2A and control RNAi are as follows: for PAIP2A shRNA, 5′-TGG GAA GAA GAA TTT ATT G-3′ (obtained from siDirect); for control shRNA, 5′-ATA TCG AGT CTT AGA CGA A-3′ (obtained scramble sequence from GenScript). The annealed shRNA cassettes were then cloned into the Mlu I and Cla I sites of the pLVTHM vector (#12247, Addgene).

### Lentiviral transduction

Lentiviruses were produced by cotransfection into LX293T cells (#632180, TaKaRa) with a transfer vector (pWPXL or pLVTHM containing a fusion protein cassette or shRNA, respectively), the packaging plasmid psPAX2, and the envelope plasmid pMD2.G (#12260 and #12259, Addgene). Titers were determined by using the qPCR Lentivirus Titration Kit (#LV900, Abm) according to the manufacturer’s protocol. Dissociated hippocampal neurons for immunoblotting or immunoprecipitation were infected with lentivirus expressing EGFP-KIF17, GFP-Ub, or GFP-Ub^K0^ at DIV 17 and incubated in lentivirus-containing media for 6 hours. After infection, lentivirus-containing media were replaced with culture media that had been used. Following transduction, the neurons were incubated for 4 to 6 days. For stimulation and localization analysis, neurons were infected with lentivirus expressing EGFP-KIF17 at DIV 17. In the case of tagRFP cotransfection, neurons were infected with lentivirus expressing tagRFP at DIV 7, and then, 10 days later, they were infected with lentivirus expressing EGFP-KIF17. For live cell imaging of KIF17 degradation, neurons were infected with lentivirus expressing PSD-95-pTagRFP at DIV 3 to 4, and then, 3 to 4 days later, they were infected with lentivirus expressing PA-GFP-KIF17. For live cell imaging of the translation reporter, neurons were infected at DIV 17 to 18 with lentivirus expressing Kaede-KIF17(−)3′UTR or Kaede-Control UTR. For live cell imaging of the newly synthesized KIF17 transport, neurons were infected at DIV 8 to 9 with lentivirus expressing mEos3.2-KIF17(+)3′UTR, mEos3.2-KIF17(ΔM)3′UTR, or mEos3.2-KIF17(ΔT)3′UTR. For live cell imaging after PAIP2A RNAi, neurons were infected at DIV 7 with shRNA against PAIP2A or control shRNA. Infected neurons for imaging were incubated in lentivirus-containing media for 4 to 6 days, except for incubation of PA-GFP-KIF17, mEos3.2-KIF17(+, ΔM, and ΔT)3′UTR, and shRNAs at 1 to 2 days, 9 to 10 days, and 14 days, respectively.

### Stimulation of hippocampal neurons

At DIV 21 to 24, hippocampal neurons were stimulated by high KCl, NMDA, or glutamate as described previously (*14*, *46*) with minor modifications. Before stimulation, neurons were incubated in Hepes-buffered saline (HBS) [110 mM NaCl, 5.4 mM KCl, 1.8 mM CaCl_2_, 0.8 mM MgCl_2_, 10 mM d-glucose, and 10 mM Hepes-NaOH (pH 7.4)] for 60 min. Neurons were stimulated for 5 min with either high KCl (same as HBS except for 55 mM NaCl and 60 mM KCl) or 25 μM glutamate (#070-00502, Wako). After stimulation, neurons were recovered in HBS for 3 to 60 min. In the case of NMDA, neurons were incubated in artificial cerebrospinal fluid (aCSF) (119 mM NaCl, 2.5 mM KCl, 1.3 mM MgSO_4_, 1.0 mM NaH_2_PO_4_, 26 mM NaHCO_3_, 2.5 mM CaCl_2_, and 11 mM d-glucose) and stimulated with Mg^2+^-free aCSF (aCSF without MgSO_4_) containing 50 μM NMDA (#132-13681, Wako). Neurons were pretreated with APV (50 μM, 50 min; #165304, Calbiochem), lactacystin A (10 μM, 15 min; #426100, Calbiochem), MG132 (10 μM, 15 min; #474790, Calbiochem), calpeptin (10 μM, 20 min; #03-34-0051, Calbiochem), caspase-3 inhibitor II (10 μM, 20 min; #264155 Calbiochem), leupeptin (100 μg/ml, 60 min; #4041, Peptide Institute Inc.), chloroquine (200 μM, 60 min; #C6628, Sigma-Aldrich), anisomycin (40 μM, 20 min; #A9789, Sigma-Aldrich), puromycin (3 μM, 15 min; #29455-54, Nacalai Tesque), actinomycin D (5 μM, 10 min; #A9415, Sigma-Aldrich), or cytochalasin D (1 μM, 30 min; #11330, Cayman Chemical). These reagents were used to antagonize NMDA receptor (APV), proteasome activity (lactacystin A and MG132), calpain activity (calpeptin), caspase-3 activity (caspase-3 inhibitor II), lysosome activity (leupeptin and chloroquine), protein synthesis (anisomycin and puromycin), and transcription (actinomycin D), respectively. Cytochalasin D was used to depolymerize the actin cytoskeleton. Antagonists remained in the incubation media for the duration of the experiments.

### Antibodies

The primary antibodies used in this study were a rabbit anti-KIF17 polyclonal antibody (pAb) [1:1000 for immunocytochemistry and 1:500 for immunoblotting, generated previously (*28*)], an anti-GFP pAb (1:1000; #598, MBL International and #A11122, Thermo Fisher Scientific), an anti-multiubiquitin monoclonal antibody (mAb) (1:1000; #D071-3, clone: FK1, MBL International), an anti–β-actin mAb (1:10,000; A-5441, Sigma-Aldrich), an anti-KIF1A pAb [1:1000; generated in our laboratory (*59*)], an anti-KIF5C pAb [1:1000; generated in our laboratory (*60*)], an anti-KIF3A mAb (1:300; #611508, BD Transduction Laboratories), anti-NR2A and anti-NR2B pAbs (1:500; #A6473 and #6474, respectively, Thermo Fisher Scientific), an anti-Mint1 mAb (1:500; #M75920, BD Transduction Laboratories), an anti-GluR1 mAb (1:500; #04-855, Upstate Biotechnology), an anti-GluR2/3 pAb (1:500; #AB1506, Millipore), an anti-PSD95 mAb (1:1000; #MA1-046, Thermo Fisher Scientific), an anti-PAIP2 pAb (1:1000; #P0087, Sigma-Aldrich), and an anti-MAP2 mouse mAb (1:1000; #M4403, Sigma-Aldrich) and chicken pAb (1:1000; #ab5392, Abcam). The Alexa Fluor 488, Alexa Fluor 568, and Alexa Fluor 647 fluorescent secondary antibodies (1:1000) were obtained from Molecular Probes, and horseradish peroxidase–conjugated secondary antibodies (1:1000) were obtained from Amersham Pharmacia. These antibodies above were used for immunoblotting and immunocytochemistry.

### Immunoblotting

For immunoblotting, stimulated or unstimulated culture neurons were fixed by 10% trichloroacetic acid/150 mM NaCl at 4°C for 30 min, detached by a cell scraper (#MS-93100 SUMILON) from a coverslip, and then centrifuged at 15,000 rpm, 4°C for 10 min. Fix solution was almost removed, and the tubes containing fixed cells were centrifuged under the same condition. Fixed cells were lysed in lithium dodecyl sulfate (LiDS) sample buffer (6.3 M urea, 1.4% Triton X-100, 2% LiDS, and 100 mM tris). Samples were mixed with dithiothreitol and boiled at 98°C for 5 min. On the other hand, fear-conditioned mouse hippocampi and mPFC were dissected by brain matrix (World Precision Instruments Inc.) and homogenized in ice-cold homogenize buffer [10 mM Hepes (pH 7.4), 0.5% NP-40, and 150 mM NaCl] containing protease inhibitor cocktail (cOmplete, Mini, EDTA-free, Roche Diagnostics). Homogenates were centrifuged at 15,000 rpm, 4°C for 15 min. Supernatants were subjected to a protein concentration assay, mixed with equal volume of 2× sample buffer [120 mM tris (pH 6.8), 20% glycerol, 4% SDS, and 0.01% bromophenol blue], and boiled at 98°C for 5 min. Equal volumes of total protein were separated by SDS–polyacrylamide gel electrophoresis, transferred onto the polyvinylidene difluoride Immobilon membrane (EMD Millipore), and then probed with specific primary antibodies and corresponding horseradish peroxidase–conjugated secondary antibodies. Detection was performed using an electrochemiluminescence procedure (#RPN2232, GE Healthcare). Band signals were quantified using ImageJ software (National Institutes of Health, Bethesda, MD).

### Immunoprecipitation

For immunoprecipitation, stimulated or unstimulated culture neurons were lysed in lysis buffer [50 mM tris (pH 7.5), 250 mM NaCl, 0.1% NP-40, 2 mM EDTA, and 10% glycerol] containing protease inhibitor cocktail (cOmplete, Mini, EDTA-free), and the supernatants were subjected to a protein concentration assay. Equal volumes of lysates were mixed with 2 μg of anti-GFP antibody (#M048-3, clone: 1E4, MBL International) conjugated with protein A–coupled Sepharose beads (nProtein A Sepharose 4 Fast Flow, GE Healthcare), and the mixture was incubated at 4°C for 48 hours with rotation. The beads were spun down at 3000 rpm for 10 s and then washed three times with immunoprecipitation buffer [150 mM NaCl, 10 mM Hepes (pH 7.4), and 0.1% Triton X-100] containing protease inhibitor cocktail. The washed beads were resuspended in 2× sample buffer and boiled at 98°C for 10 min, following the same procedure as for immunoblotting.

### Immunocytochemistry

Stimulated and unstimulated culture neurons (DIV 21 to 24) were washed once in phosphate-buffered saline (PBS) and fixed with 4% PFA in PBS at 37°C for 10 min. Fixed neurons were permeabilized with 0.1% Triton X-100 in PBS and blocked with PBS supplemented with 5% bovine serum albumin for 20 min, and primary antibodies were incubated at 4°C overnight, followed by the corresponding Alexa Fluor-labeled antibodies (Alexa Fluor 488, Alexa Fluor 568, or Alexa Fluor 647; Molecular Probes) at room temperature (R/T) for 1 hour. Images were acquired using an LSM780 confocal laser scanning microscope. The images were quantified using MetaMorph (Molecular Devices) and ImageJ software.

### Immunohistochemistry

AAV-injected mice (*Kif17* 3′UTR*^2lox/2lox^*: GFP and *Kif17* 3’UTR*^2lox/2lox^*: Cre-GFP mice) were anesthetized and subjected to perfusion fixation with 4% PFA in 0.1 M K-phosphate buffer 3 weeks after the vector infusion. The fixed brains were permeated with sucrose, embedded in O.C.T. Compound (Sakura Finetek), frozen with liquid N_2_, and cryosectioned with a Leica CM1950 microtome at 30 μm thick. The sections were washed with PBT (0.5% Tween 20 in PBS), blocked with 5% normal goat serum in PBT at R/T for 1 hour, and incubated with primary antibodies against KIF17 (1:200), NR2B (1:200), or phospho-CREB (Ser^133^) (1:200; #9198, Cell Signaling Technology) diluted in the blocking buffer at 4°C overnight, followed by the corresponding Alexa Fluor–conjugated secondary antibodies (1:200) at R/T for 3 hours. In the case of immunohistostaining for phospho-CREB, 4′,6-diamidino-2-phenylindole solution staining (1:1000; 340-07971, Dojindo Laboratories) was simultaneously performed. For the immunohistochemical detection of NR2B or phospho-CREB, sections were treated with pepsin (1.0 mg/ml; #12979-82, Nacalai Tesque) in 0.2 N HCl at 37°C for 10 min or 0.5% H_2_O_2_ in tris-buffered saline (pH 7.4) at R/T for 30 min, respectively, followed by the blocking. The stained sections were observed on an LSM780 confocal laser scanning microscope.

### Live cell imaging

Before imaging, neurons were incubated in HBS or aCSF for 60 min, and then, the neurons were globally illuminated for photoactivation and photoconversion by a 405-nm laser within an LSM780 confocal laser scanning microscope. The laser illuminated the neurons at 5 to 8% of full power for 1.5 to 2.0 min. When needed, neurons were pretreated with lactacystin A (10 μM, 15 min), anisomycin (40 μM, 20 min), actinomycin D (5 μM, 10 min), calpeptin (10 μM, 20 min), or cytochalasin D (1 μM, 30 min). For live cell imaging of KIF17 degradation and transport of the newly synthesized KIF17, stimulation was carried out by applying NMDA (50 and 20 μM, respectively) in Mg^2+^-free aCSF. Images were taken by an LSM780 microscope every 1.5 to 2.0 s for 12 min at most starting 1 to 3 min after NMDA application. For live cell imaging of the translation reporter, stimulation was carried out by applying NMDA (50 μM) in Mg^2+^-free aCSF, glutamate (25 μM) in HBS, or high KCl (60 mM) for 5 min, followed by washout with aCSF or HBS (containing antagonists when needed). Images were taken by an LSM780 microscope every 1.5 to 3.5 min for 60 min starting 1 to 3 min after the end of stimulation. For monitoring Kaede and mEos3.2 syntheses, the green and red forms were imaged simultaneously, which showed that stimulation did not induce any changes in the red channel. Neurite transection was performed by the full power of a 405-nm laser operated with an LSM780 microscope for 10 to 15 min. Proximal dendrites at 30 to 60 μm from cell bodies were illuminated, avoiding any branch points. The Kaede imaging was started immediately after the laser transection was completed. Imaging was performed at 37°C with 5% CO_2_. The images were quantified using MetaMorph and ImageJ software.

### Quantitative reverse-transcription polymerase chain reaction

Total RNA was isolated from cultured neurons using an Agilent Total RNA Isolation Mini Kit (#5185-6000, Agilent Technologies) according to the manufacturer’s protocol. Then, cDNA was produced from 1 μg of total RNA using a First-strand cDNA Synthesis System for Quantitative RT-PCR (#11801, Marligen Biosciences Inc.) according to the manufacturer’s protocol. The cDNA samples were subjected to amplification with a GeneAce SYBR qPCR Mix α No ROX (#319-07703, NIPPON GENE) in a LightCycler 480 system (Roche) with preincubation at 95°C for 30 s, denaturation at 95°C for 10 s, annealing at 55° to 56°C for 15 s, and extension step at 72°C for 30 s for 40 cycles. The program also included a final extension at 72°C for 10 min. The PCR primers were as follows: for *Kif17*, 5′-GTT TGA GAG AGC GTT CAG TGT GC-3′ and 5′-GGA TGC TCC TTC AGC TCA AG-3′; for *Kif5c*, 5′-GGC TGG GAG TGA AAA GGT CAG-3′ and 5′-CCG GTA CAT GTG TTT TTG TCC C-3′; for Arc, 5′-CCA GGA GAA TGA CAC CAG GTC-3′ and 5′-GGA ACC CAT GTA GGC AGC TT-3′; and for β*-actin*, 5′-GCA CCA CAC CTT CTA CAA TGA G-3′ and 5′-GAA GGT CTC AAA CAT GAT CTG G-3′. The results from β*-actin* were treated as an internal control.

### In situ hybridization

#### Fluorescence in situ hybridization

In situ hybridization was performed using digoxigenin-labeled antisense riboprobes. A fragment (619 to 1422 bp from the start codon) of KIF17 cDNA was subcloned into the Eco RI and Sal I sites of pBluescript II KS− (Stratagene). The antisense and sense probes were transcribed in the presence of digoxigenin-RNA mix (#11277073910, Roche) using T7 polymerase (#10881767001, Roche) and T3 polymerase (#11031163001, Roche), respectively. Culture neurons (DIV 21) were fixed with 4% PFA in PBS at R/T for 15 min, permeabilized with 0.1% Triton X-100 in PBS at R/T for 5 min, and equilibrated with 5× SSC at R/T for 15 min. Fixed neurons were then prehybridized for 2 hours at 58°C with prehybridization mix [50% formamide, 5× SSC, and salmon sperm DNA (0.1 mg/ml)]. The probes were denatured at 80°C for 5 min and added to the hybridization mix (1:100). The hybridization reaction was performed at 58°C for 40 hours. Prehybridization and hybridization were performed in a box saturated with a 5× SSC and 50% formamide solution to avoid evaporation. Following incubation, the cultured neurons were washed three times at 58°C for 30 min with 2× SSC, followed by two washes at R/T for 30 min with 0.1× SSC, and equilibrated at R/T for 5 min with buffer 1 [100 mM tris (pH 7.5) and 150 mM NaCl]. The fixed neurons were then incubated at R/T for 2 hours with anti-digoxigenin antibody (1:2000) (#11333089001, Roche) and anti-MAP2 antibody (1:1000) diluted in buffer 1 containing 1% blocking reagent, followed by the corresponding Alexa Fluor-labeled antibodies. Images were acquired using an LSM780 confocal laser scanning microscope.

#### Quantitative FISH

Custom Stellaris FISH Probes were designed against Kif17 mRNA containing 3′UTR by using the Stellaris FISH Probe Designer (LGC Biosearch Technologies Inc.) available online at www.biosearchtech.com/stellarisdesigner. The dissociated hippocampal culture neurons (DIV 21 to 23) and coronally cryosectioned brains were hybridized with the Kif17 mRNA containing 3′UTR Stellaris FISH probe set labeled with Quasar 570 (Biosearch Technologies Inc.), following the manufacturer’s instructions available online at www.biosearchtech.com/support/resources/stellaris-protocols. Stellaris FISH Probe recognizing mouse Gapdh transcripts (the coding sequence of NM_008084.2 nts 51-1052) labeled with Quasar 570 (#SMF-3002-1, LGC Biosearch Technologies Inc., Petaluma, CA) were used as controls. Briefly, neurons were fixed with 4% PFA in PBS for 10 min at 37°C, rinsed twice with PBS, and permeabilized with 70% (vol/vol) ethanol at 4°C for longer than an hour. The neurons were then washed with Stellaris RNA FISH Wash Buffer A (#SMF-WA1-60, LGC Biosearch Technologies Inc.) at R/T for 5 min. Hybridization buffer (#SMF-HB1-10, LGC Biosearch Technologies Inc.) was used to dilute probes and an anti-MAP2 chicken antibody and applied to the neurons at 37°C for 16 hours in the dark. Hybridized neurons were then washed with Wash Buffer A containing Alexa Fluor 488–conjugated anti-chicken antibody at 37°C for an hour in the dark. Last, the neurons were washed with Stellaris RNA FISH Wash Buffer B (#SMF-WA1-60, LGC Biosearch Technologies Inc.) at R/T for 5 min and subjected to the image acquisition. Signal dots along MAP2-positive dendrites of acquired images were counted using ImageJ.

### Proximity ligation assay

For the PLA, hippocampal culture neurons (DIV 21) treated with puromycin were fixed with PFA-sucrose (4% PFA, 4% sucrose in PBS) at 37°C for 10 min. Fixed neurons were initially processed for immunocytochemistry as described above and incubated with a rabbit anti-KIF17 antibody (1:500) and a mouse anti-puromycin antibody (1:500; #PEN-MA001, clone: 3RH11, Cosmo Bio Co. Ltd.) at 4°C overnight. Then, they were subjected to a Duolink in situ PLA kit (Sigma-Aldrich) according to the manufacturer’s protocols and observed with an LSM780 confocal laser scanning microscope. The number of signal puncta in a single soma and a 100-μm span of dendritic shafts was manually counted and compared.

### Statistical analyses

All data are presented as the means ± SEM except for the box plots in Fig. 7A and fig. S9. Two-tailed Student’s *t* test, paired *t* test, or Welch *t* test was used to compare two independent samples when appropriate. One-way analysis of variance (ANOVA) or two-way ANOVA with Bonferroni’s post hoc test was used for multiple comparisons. The significance level was set at 0.05. The Smirnov-Grubbs’ test was used for evaluating outliers.

## Supplementary Material

http://advances.sciencemag.org/cgi/content/full/6/51/eabc8355/DC1

Movie S1

Movie S2

Movie S3

Movie S4

Movie S5

Movie S6

Movie S7

Adobe PDF - abc8355_SM.pdf

An activity-dependent local transport regulation via degradation and synthesis of KIF17 underlying cognitive flexibility

## SUPPLEMENTARY MATERIALS

Supplementary material for this article is available at http://advances.sciencemag.org/cgi/content/full/6/51/eabc8355/DC1

## Appendix

View/request a protocol for this paper from *Bio-protocol*.
