# Supplementary material for: An activity-dependent local transport regulation via degradation and synthesis of KIF17 underlying cognitive flexibility
Source: Sci Adv. 2020 Dec 16;6(51):eabc8355. doi: 10.1126/sciadv.abc8355 (PMC7744090; doi:10.1126/sciadv.abc8355)
Supplement: http://advances.sciencemag.org/cgi/content/full/6/51/eabc8355/DC1 [file supp_6_51_eabc8355__index.html]

Science Advances | Science AdvancesAAASSearchScience AdvancesMenu

## Supplementary Materials

# An activity-dependent local transport regulation via degradation and synthesis of KIF17 underlying cognitive flexibility

Suguru Iwata, Momo Morikawa, Yosuke Takei, Nobutaka Hirokawa

Download Supplement

**The PDF file includes:**

- Figs. S1 to S10
- Legends for movies S1 to S7

**Other Supplementary Material for this manuscript includes the following:**

- Movie S1
- Movie S2
- Movie S3
- Movie S4
- Movie S5
- Movie S6
- Movie S7

**Files in this Data Supplement:**

- Adobe PDF - abc8355\_SM.pdf
- abc8355\_Movie\_S1.avi
- abc8355\_Movie\_S2.avi
- abc8355\_Movie\_S3.avi
- abc8355\_Movie\_S4.avi
- abc8355\_Movie\_S5.avi
- abc8355\_Movie\_S6.avi
- abc8355\_Movie\_S7.avi
