# Supplementary material for: An activity-dependent local transport regulation via degradation and synthesis of KIF17 underlying cognitive flexibility
Source: Sci Adv. 2020 Dec 16;6(51):eabc8355. doi: 10.1126/sciadv.abc8355 (PMC7744090; doi:10.1126/sciadv.abc8355)
Supplement: http://advances.sciencemag.org/cgi/content/full/6/51/eabc8355/DC1 [file supp_6_51_eabc8355__1.pdf]

Science Advances
AAAS

advances.sciencemag.org/cgi/content/full/6/51/eabc8355/DC1

Supplementary Materials for

An activity-dependent local transport regulation via degradation and synthesis of KIF17 underlying cognitive flexibility

Suguru Iwata, Momo Morikawa, Yosuke Takei, Nobutaka Hirokawa*

*Corresponding author. Email: hirokawa@m.u-tokyo.ac.jp

Published 16 December 2020, *Sci. Adv.* **6**, eabc8355 (2020)
DOI: 10.1126/sciadv.abc8355

The PDF file includes:

Figs. S1 to S10
Legends for movies S1 to S7

Other Supplementary Material for this manuscript includes the following:

(available at advances.sciencemag.org/cgi/content/full/6/51/eabc8355/DC1)

Movies S1 to S7

Supplementary Figures

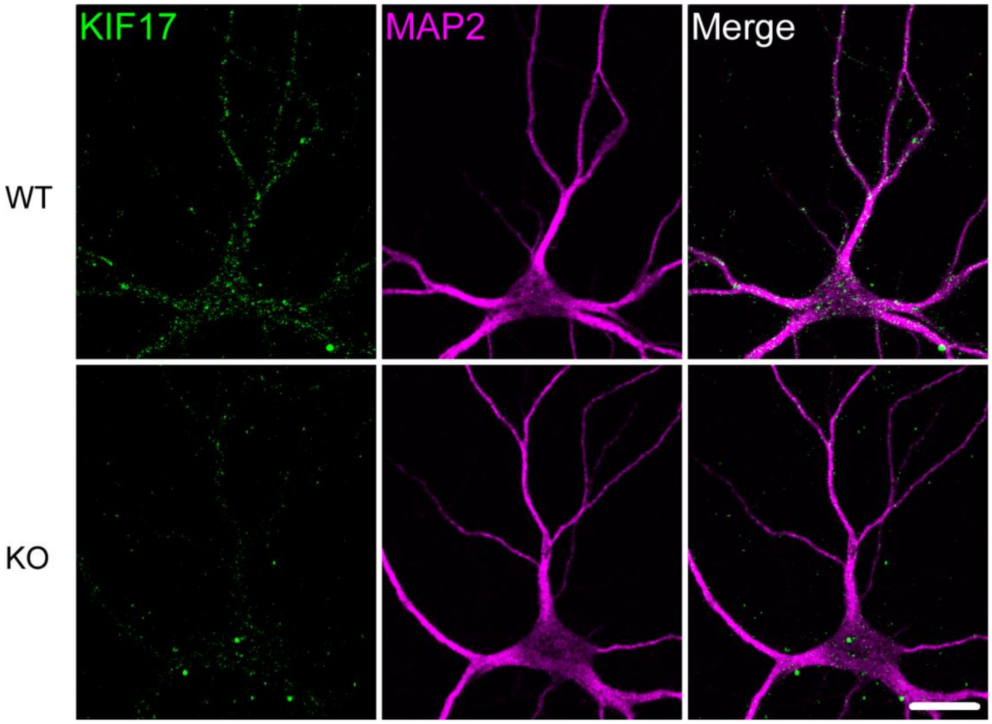

Fig. S1, related to Fig. 1. KIF17 is abundantly expressed in somatic and dendritic compartments of hippocampal culture neurons. Immunofluorescence cytochemistry of hippocampal culture neurons (DIV 21) from WT (*kif17*$^{+/+}$) and KO (*kif17*$^{-/-}$) mice using an anti-KIF17 antibody and an anti-MAP2 antibody. Scale bar, 20 μm.

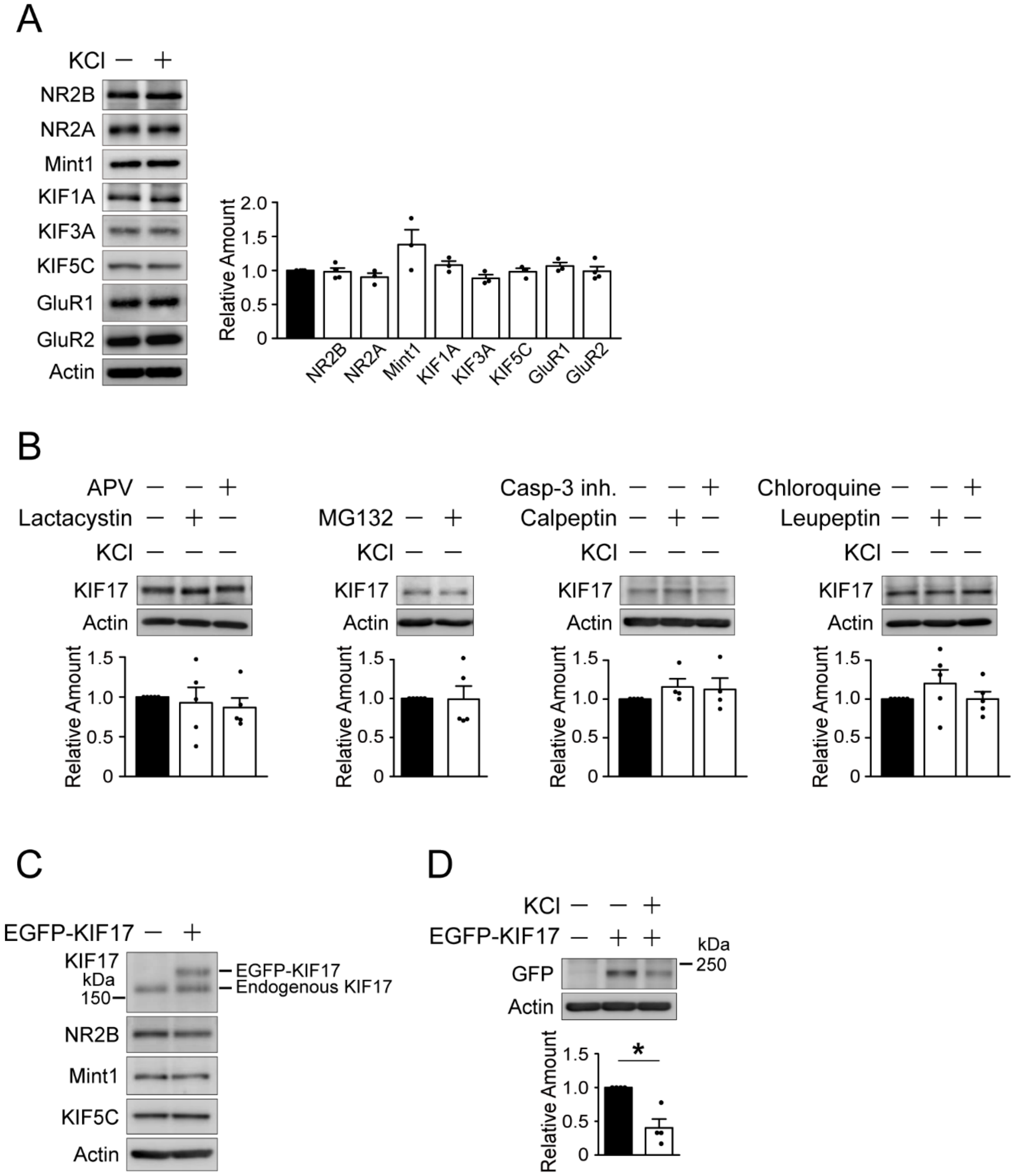

A
KCl
NR2B
NR2A
Mint1
KIF1A
KIF3A
KIF5C
GluR1
GluR2
Actin
Relative Amount
B
APV
Lactacystin
KCl
KIF17
Actin
MG132
Casp-3 inh.
Calpeptin
Chloroquine
Leupeptin
C
EGFP-KIF17
kDa
150
EGFP-KIF17
Endogenous KIF17
D
GFP
250
*

Fig. S2, related to Fig. 1. Supplemental immunoblots of the lysates from dissociated hippocampal neurons. (A) There were no changes in the levels of the indicated KIF17-related proteins when neurons (DIV 21–24) were stimulated with high-KCl. Data are expressed as the mean ± SEM throughout the supplemental figures unless otherwise mentioned. $p \geq 0.05$, one-way ANOVA. n = 3–4 independent experiments. **(B)** Effects on KIF17 levels following the application of the indicated reagents without high-KCl stimulation. Note that lactacystin A and APV, MG132, calpeptin and caspase-3 inhibitor, and leupeptin and chloroquine treatments without high-KCl stimulation had no significant effects on KIF17 expression. $p \geq 0.05$, one-way ANOVA and two-tailed *t* test. n = 4–5 independent experiments. **(C)** Comparison of the endogenous KIF17 levels and the EGFP-KIF17 levels in the neurons expressing EGFP-KIF17. This overexpression had no significant effects on the expression of the proteins related to KIF17 (NR2B, Mint1, and KIF5C). **(D)** Degradation of EGFP-KIF17 is induced by high-KCl-induced stimulation. $^*p < 0.05$, two-tailed *t* test. n = 4 independent experiments.

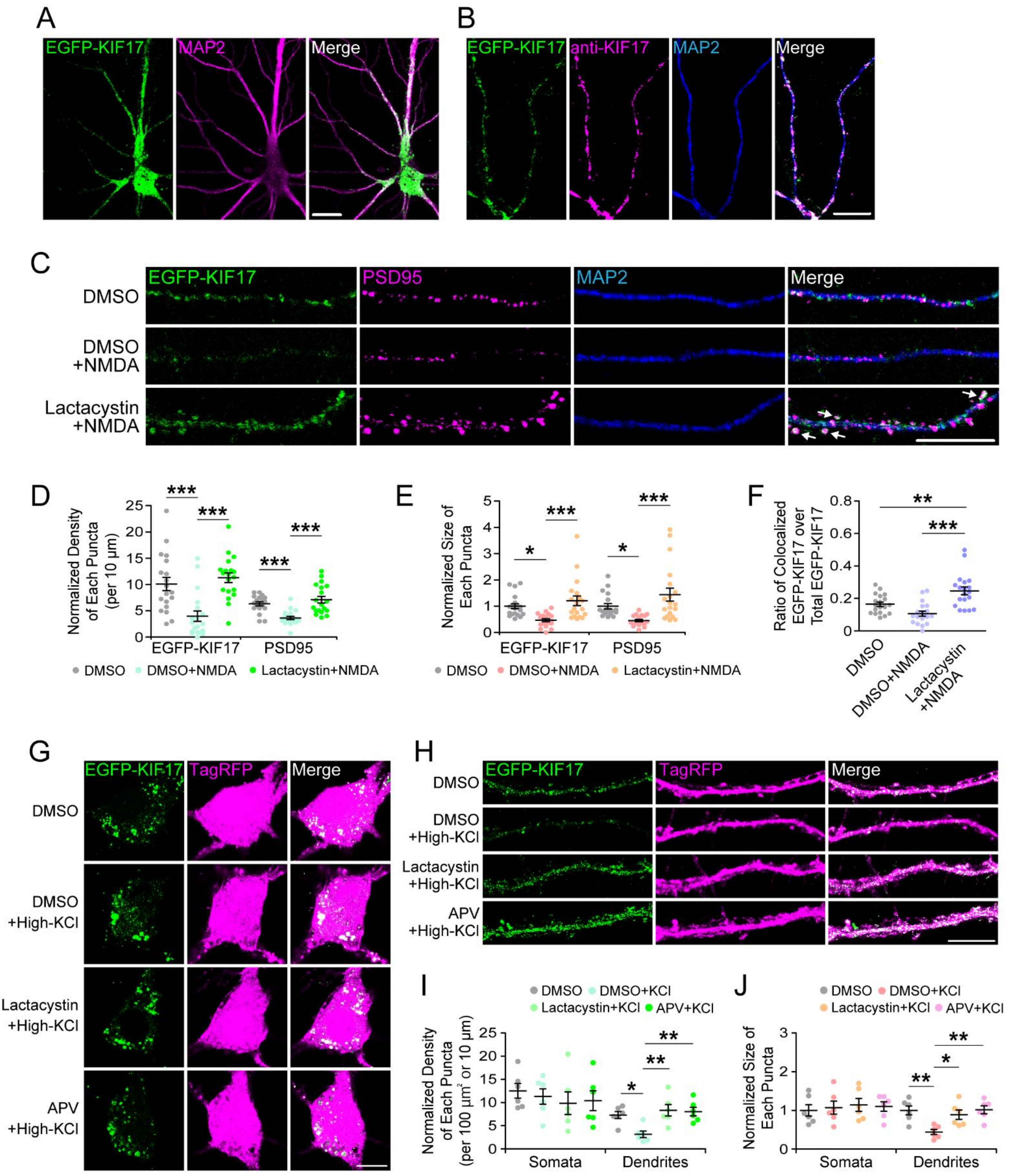
A
EGFP-KIF17
MAP2
Merge
B
EGFP-KIF17
anti-KIF17
MAP2
Merge
C
EGFP-KIF17
PSD95
MAP2
Merge
DMSO
DMSO +NMDA
Lactacystin +NMDA
D
Normalized Density of Each Puncta (per 10 μm)
EGFP-KIF17
PSD95
DMSO
DMSO+NMDA
Lactacystin+NMDA
E
Normalized Size of Each Puncta
EGFP-KIF17
PSD95
DMSO
DMSO+NMDA
Lactacystin+NMDA
F
Ratio of Colocalized EGFP-KIF17 over Total EGFP-KIF17
DMSO
DMSO+NMDA
Lactacystin +NMDA
G
EGFP-KIF17
TagRFP
Merge
DMSO
DMSO +High-KCl
Lactacystin +High-KCl
APV +High-KCl
H
EGFP-KIF17
TagRFP
Merge
DMSO
DMSO +High-KCl
Lactacystin +High-KCl
APV +High-KCl
I
DMSO
DMSO+KCl
Lactacystin+KCl
APV+KCl
Normalized Density of Each Puncta (per 100 μm² or 10 μm)
Somata
Dendrites
J
DMSO
DMSO+KCl
Lactacystin+KCl
APV+KCl
Normalized Size of Each Puncta
Somata
Dendrites

Fig. S3, related to Fig. 2. Supplemental experiments related to the local degradation of KIF17. (A) Images of mouse hippocampal neurons expressing EGFP-KIF17 (DIV 21). The neurons were immunostained with an antibody against MAP2, a shaft marker of dendrites. EGFP-KIF17 was localized at dendritic shafts. Scale bar, 20 µm. **(B)** Images of dendrites expressing EGFP-KIF17 (DIV 21). The neurons were immunostained with anti-KIF17 and anti-MAP2 antibodies. Anti-KIF17 positive puncta showed 64.9 ± 3.2% colocalization with the EGFP-KIF17 puncta. A total of 969 anti-KIF17 positive puncta were assessed from 18 neurons. Scale bar, 10 µm. **(C)** Representative images of dendrites expressing EGFP-KIF17. The neurons were treated as indicated and immunostained with anti-PSD95 and anti-MAP2 antibodies. Scale bar, 10 µm. **(D** and **E)** Quantification of the normalized density (D) and size (E) of the EGFP-KIF17 and anti-PSD95 positive puncta. Note that the density and size of each puncta were significantly decreased upon NMDA-induced stimulation (DMSO+NMDA), which was rescued by lactacystin A (Lactacystin+NMDA). $p < 0.001$, one-way ANOVA; $*p < 0.05$, $***p < 0.001$, Bonferroni's post hoc comparison. n = 19 dendrites from four independent cultures. **(F)** The ratio of the EGFP-KIF17 puncta colocalized with the anti-PSD95 positive puncta over the total EGFP-KIF17 puncta. Note that the colocalization ratio increased in the Lactacystin+NMDA dendrites compared with the DMSO and DMSO+NMDA controls. $p < 0.001$, one-way ANOVA; $**p < 0.01$, $***p < 0.001$, Bonferroni's post hoc test. n = 19 dendrites from four independent cultures. **(G** to **J)** Representative images of somata (G) and dendrites (H) expressing EGFP-KIF17 and TagRFP, and the quantifications of the normalized density (I) and size (J) of the EGFP-KIF17 puncta in somata and dendrites. The density and size of EGFP-KIF17 puncta were significantly decreased upon high-KCl-induced stimulation in dendrites but not in somata. Scale bar, 10 µm. $p < 0.01$, one-way ANOVA; $*p < 0.05$, $**p < 0.01$, Bonferroni's post hoc comparison. n = 6 somata and dendrites from four independent cultures.

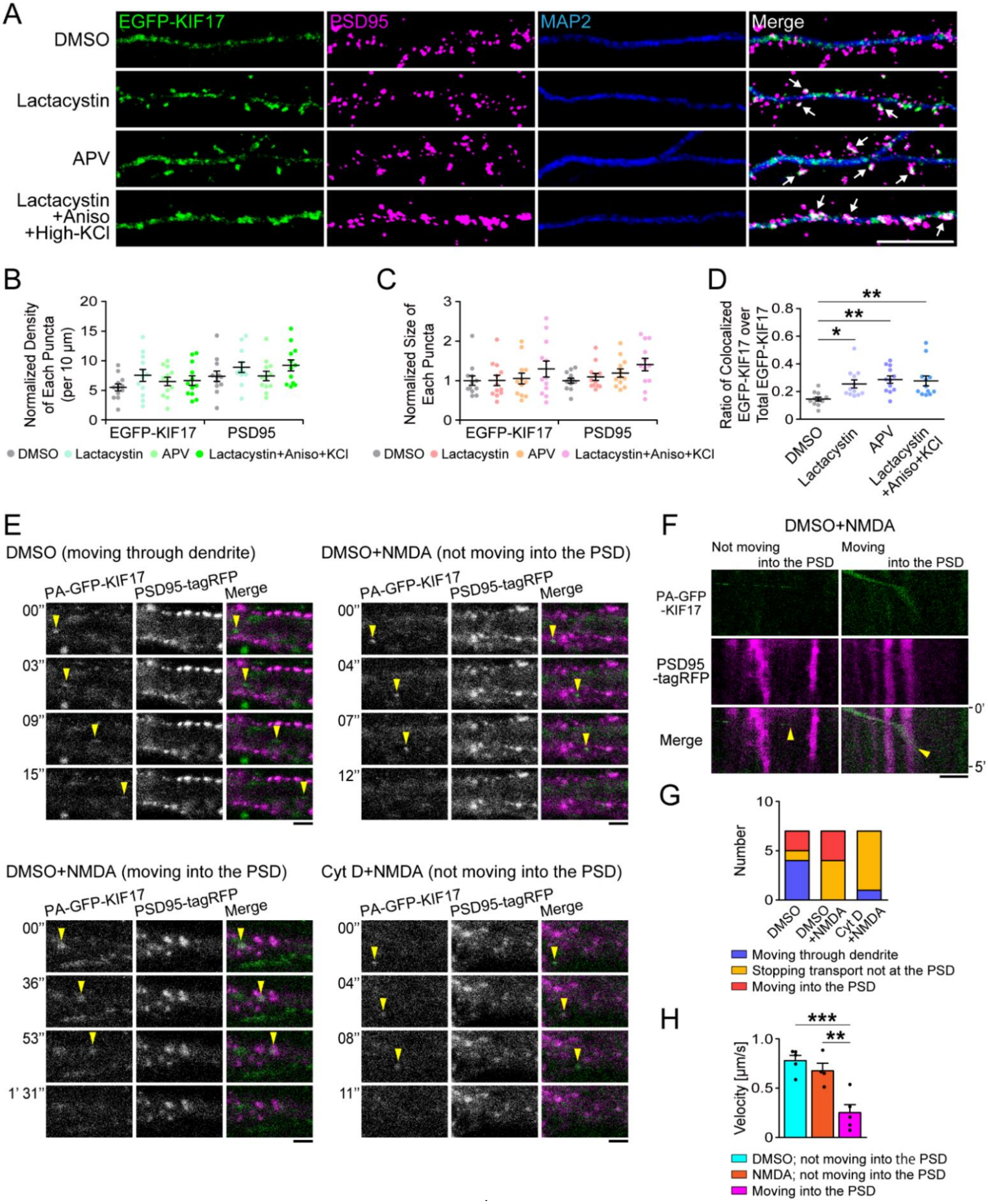

A
EGFP-KIF17
PSD95
MAP2
Merge
DMSO
Lactacystin
APV
Lactacystin +Aniso +High-KCl
B
Normalized Density of Each Puncta (per 10 μm)
EGFP-KIF17
PSD95
DMSO
Lactacystin
APV
Lactacystin+Aniso+KCl
C
Normalized Size of Each Puncta
EGFP-KIF17
PSD95
DMSO
Lactacystin
APV
Lactacystin+Aniso+KCl
D
Ratio of Colocalized EGFP-KIF17 over Total EGFP-KIF17
DMSO
Lactacystin
APV
Lactacystin +Aniso+KCl
E
DMSO (moving through dendrite)
DMSO+NMDA (not moving into the PSD)
DMSO+NMDA (moving into the PSD)
Cyt D+NMDA (not moving into the PSD)
PA-GFP-KIF17
PSD95-tagRFP
Merge
F
DMSO+NMDA
Not moving into the PSD
Moving into the PSD
PA-GFP -KIF17
PSD95 -tagRFP
Merge
G
Number
DMSO
DMSO +NMDA
Cyt D +NMDA
Moving through dendrite
Stopping transport not at the PSD
Moving into the PSD
H
Velocity [μm/s]
DMSO; not moving into the PSD
NMDA; not moving into the PSD
Moving into the PSD

Fig. S4, related to Fig. 2. Supplemental experiments related to the localization of KIF17 degradation. (A) Representative images of dendrites expressing EGFP-KIF17. The neurons were treated as indicated and immunostained with anti-PSD95 and anti-MAP2 antibodies. Aniso, anisomycin. Scale bar, 10 μm. **(B** and **C)** Quantification of the normalized density (B) and size (C) of the EGFP-KIF17 and anti-PSD95 positive puncta. The density and size of each puncta did not change following application of only the indicated reagents. $p \geq 0.05$, one-way ANOVA. n = 12 dendrites from four independent cultures. **(D)** The ratio of the EGFP-KIF17 puncta colocalized with the anti-PSD95 positive puncta over the total EGFP-KIF17 puncta. Note that the colocalization ratio increased in the Lactacystin, APV, and Lactacystin+Anisomycin+KCl groups compared with the DMSO group. $p < 0.01$, one-way ANOVA; $*p < 0.05$, $**p < 0.01$, Bonferroni's post hoc test. n = 12 dendrites from four independent cultures. **(E** and **F)** Time-lapse images of PA-GFP-KIF17 and PSD95-tagRFP along dendrites (E), and the kymographs of dendrites treated with DMSO+NMDA (F). Without stimulation, the PA-GFP-KIF17 puncta tended to move through the dendrites (upper left panel), while in response to stimulation, the PA-GFP-KIF17 puncta stopped moving and disappeared. Some PA-GFP-KIF17 puncta did not move into the PSD95-tagRFP clusters (upper right panel), and the others moved into the PSD95-tagRFP clusters (lower left panel). The PA-GFP-KIF17 puncta did not move into the PSD95-tagRFP clusters in stimulated dendrites pretreated with cytochalasin D (lower right panel). Cyt D, cytochalasin D. The yellow arrowheads, PA-GFP-KIF17 (E) or the time and location of PA-GFP-KIF17 degradation (F). Scale bars, 2 μm. **(G)** Classification of the behavior of PA-GFP-KIF17 in dendrites. **(H)** Velocities for the PA-GFP-KIF17 movements. The velocity for the PA-GFP-KIF17 puncta that moved into the PSD95-tagRFP clusters significantly decreased. $p < 0.001$, one-way ANOVA; $**p < 0.01$, $***p < 0.001$, Bonferroni's post hoc comparison. DMSO; not moving into the PSD and Moving into the PSD, n = 5 moving puncta from five culture neurons; NMDA; not moving into the PSD, n = 4 moving puncta from four culture neurons.

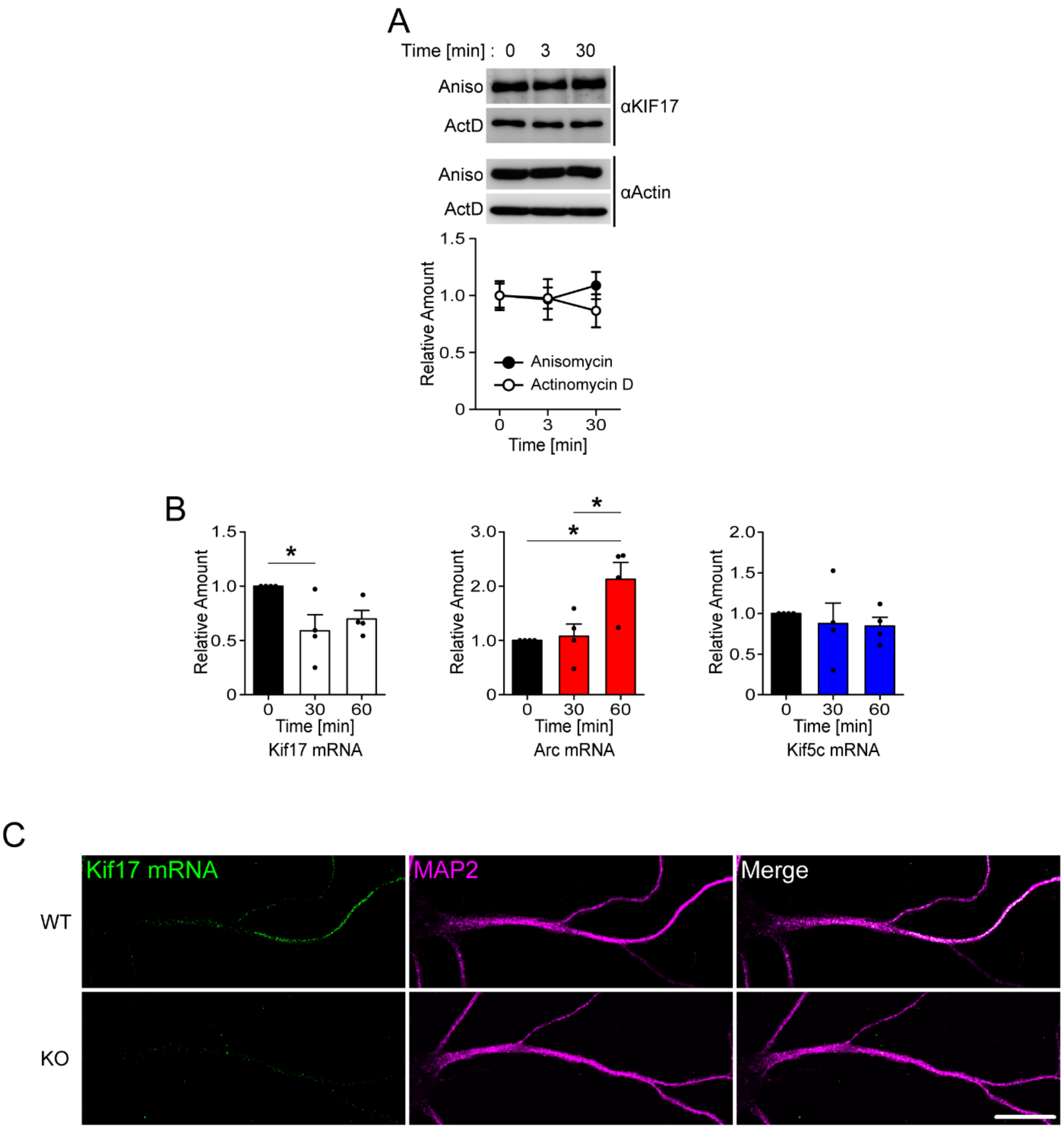

A
Time [min] : 0 3 30
Aniso
ActD
αKIF17
Aniso
ActD
αActin
Relative Amount
1.5
1.0
0.5
0
Anisomycin
Actinomycin D
0 3 30
Time [min]
B
Relative Amount
1.5
1.0
0.5
0
*
0 30 60
Time [min]
Kif17 mRNA
3.0
2.0
1.0
0
*
*
0 30 60
Time [min]
Arc mRNA
2.0
1.5
1.0
0.5
0
0 30 60
Time [min]
Kif5c mRNA
C
Kif17 mRNA
MAP2
Merge
WT
KO

Fig. S5, related to Fig. 3. Supplemental immunoblots, qRT-PCR and control for *in situ* hybridization. (A) Time courses of KIF17 expression in unstimulated neurons treated with anisomycin or actinomycin D. The levels of KIF17 did not significantly change when each of them was applied to hippocampal neurons (DIV 21–24). Aniso, anisomycin; ActD, actinomycin D. $p \geq 0.05$, one-way ANOVA. n = 5 independent experiments. **(B)** qRT-PCR analyses of the Kif17, Arc, and Kif5c mRNA levels in hippocampal neurons (DIV 21–24) normalized to the β-actin mRNA levels. The Kif17 mRNA levels decreased significantly within 30 min after high-KCl-induced stimulation. However, the Arc mRNA levels increased continuously until 60 min and the Kif5c mRNA levels did not change throughout the experiments. $p < 0.05$ (Kif17 and Arc mRNA), $p \geq 0.05$ (Kif5c mRNA), one-way ANOVA; $*p < 0.05$, Bonferroni's post hoc comparison. n = 4 independent experiments. **(C)** *In situ* hybridizations performed with *kif17* antisense riboprobes in WT ($kif17^{+/+}$) and KO ($kif17^{-/-}$) hippocampal neurons (DIV 21). Neurons were immunostained for the dendritic shaft marker MAP2. Kif17 mRNA was detected in the $kif17^{+/+}$ dendrites but not in the $kif17^{-/-}$ dendrites. Scale bar, 20 μm.

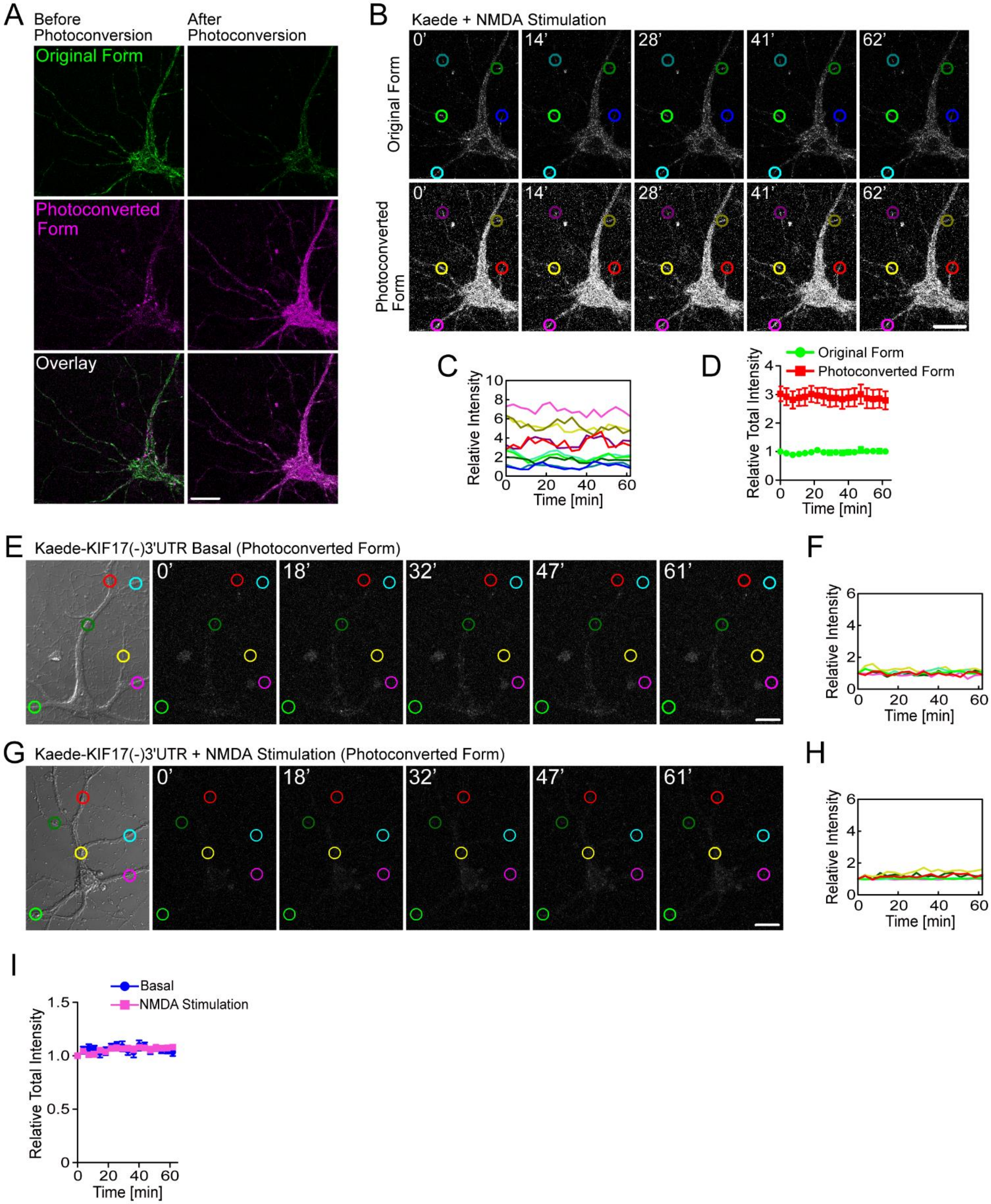

A
Before Photoconversion
After Photoconversion
Original Form
Photoconverted Form
Overlay
B
Kaede + NMDA Stimulation
0'
14'
28'
41'
62'
Original Form
Photoconverted Form
C
Relative Intensity
Time [min]
D
Original Form
Photoconverted Form
Relative Total Intensity
Time [min]
E
Kaede-KIF17(-)3'UTR Basal (Photoconverted Form)
0'
18'
32'
47'
61'
F
Relative Intensity
Time [min]
G
Kaede-KIF17(-)3'UTR + NMDA Stimulation (Photoconverted Form)
0'
18'
32'
47'
61'
H
Relative Intensity
Time [min]
I
Basal
NMDA Stimulation
Relative Total Intensity
Time [min]

Fig. S6, related to Fig. 4. Supplemental experiments related to the localized synthesis of Kaede driven by KIF17 3'UTR. (A) Kaede proteins were uniformly expressed in hippocampal culture neurons (DIV 21) transduced with a lentiviral construct containing Kaede. Illuminated by a 405 nm laser, Kaede proteins were photoconverted from green to red. Scale bar, 20 μm. **(B** to **D)** Time-lapse images of Kaede upon NMDA stimulation (B), and their temporal variations in the individual (C) and total (D) relative fluorescence intensity of Kaede. Stimulation with NMDA did not induce local synthesis of Kaede without the KIF17 3'UTR. Each five puncta in (B) were randomly chosen, and the circles' colors of the measured area correspond to the line colors in (C). The total intensity of the original (green line) and photoconverted (red line) form of Kaede did not change throughout the experiments. Scale bar, 20 μm. $p \geq 0.05$, one-way ANOVA. n = 6 neurons from four independent cultures. **(E** to **I)** Time-lapse images demonstrating the photoconverted form of Kaede upon NMDA stimulation (E and G), and their temporal variations in the individual (F and H) and total (I) relative intensity of Kaede. Each six measured area in (E) and (G) were randomly chosen, and the circles' colors of the measured area correspond to the line colors in (F) and (H). The total intensity under basal conditions (blue line) and upon NMDA stimulation (pink line) did not change throughout the experiments. The original form of Kaede is represented in Fig. 4, A and C, respectively. Scale bars, 20 μm. $p \geq 0.05$, one-way ANOVA. n = 6 neurons from four independent cultures.

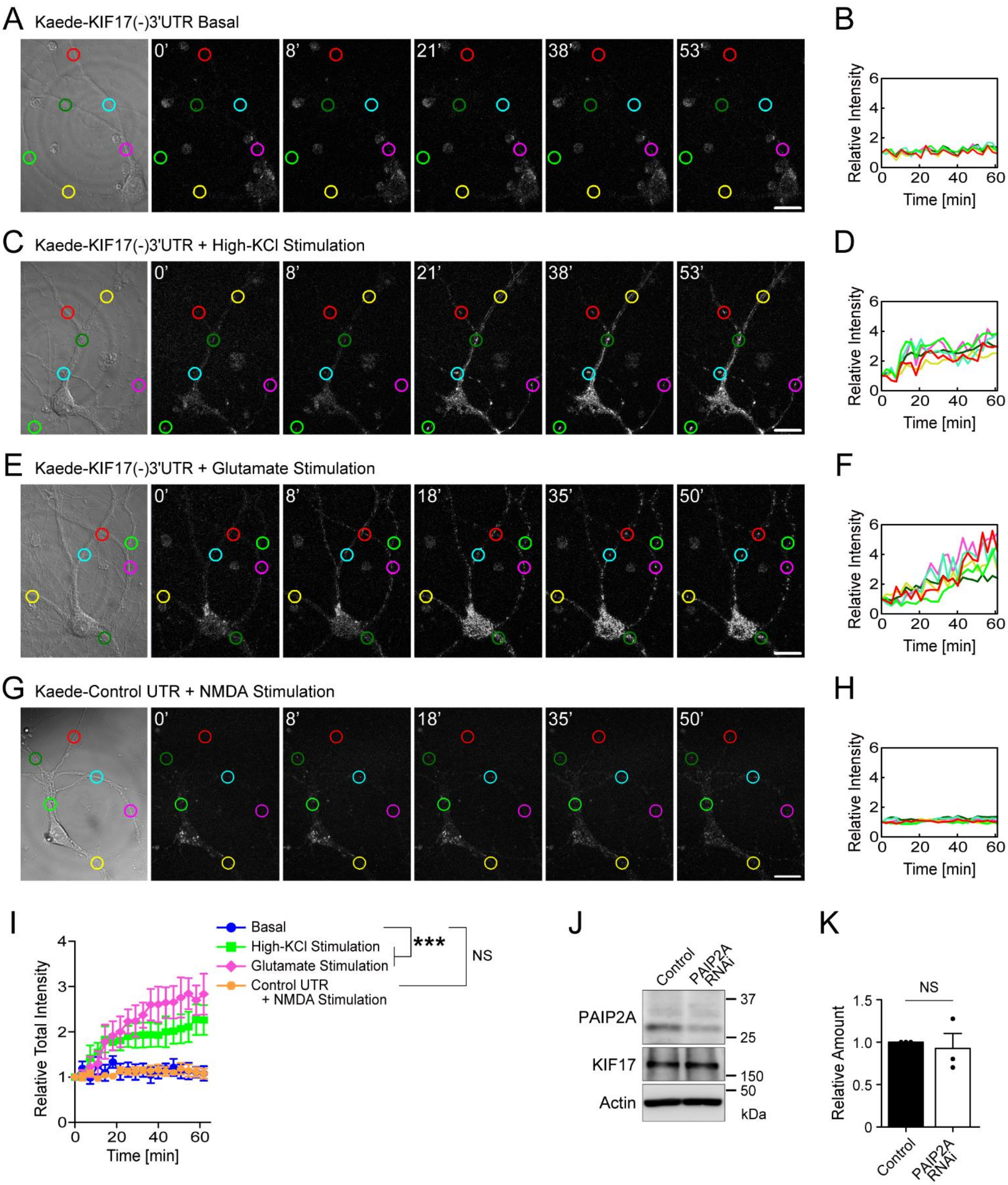
A
Kaede-KIF17(-)3'UTR Basal
0'
8'
21'
38'
53'
B
Relative Intensity
Time [min]
C
Kaede-KIF17(-)3'UTR + High-KCl Stimulation
D
E
Kaede-KIF17(-)3'UTR + Glutamate Stimulation
18'
35'
50'
F
G
Kaede-Control UTR + NMDA Stimulation
H
I
Basal
High-KCl Stimulation
Glutamate Stimulation
Control UTR
+ NMDA Stimulation
***
NS
Relative Total Intensity
J
Control
PAIP2A RNAi
PAIP2A
KIF17
Actin
37
25
150
50
kDa
K
NS
Relative Amount

Fig. S7, related to Figs. 4 and 5. Supplemental experiments for induction of localized Kaede synthesis driven by KIF17 3'UTR and assessment of the efficiency of PAIP2A shRNA. (A to **I)** Time-lapse images demonstrating the local synthesis of Kaede driven by KIF17 3'UTR (A, C, E, and G), and their temporal variations in the individual (B, D, F, and H) and total (I) relative fluorescence intensity of Kaede. Stimulations with high-KCl and glutamate induced KIF17 3'UTR-mediated local synthesis of the translation reporter Kaede. In response to stimulation, Kaede puncta were synthesized (C and E). Without stimulation, Kaede puncta were not synthesized (A). NMDA stimulation did not induce the local synthesis of Kaede driven by control UTR (G). Each six measured area in (A), (C), (E), and (G) were randomly chosen, and the circles' colors of the measured area correspond to the line colors in (B), (D), (F), and (H), respectively. Scale bars, 20 μm. Effects of treatment (***$p < 0.001$; NS, $p \geq 0.05$) and time ($p < 0.001$), two-way ANOVA. n = 3–4 neurons from three independent cultures. **(J)** Assessment of the efficiency of PAIP2A shRNA. RNAi knockdown of PAIP2A did not change the KIF17 protein levels in immunoblotting. **(K)** qRT-PCR analysis of the Kif17 mRNA levels in hippocampal neurons (DIV 21) transfected with shRNA against PAIP2A. RNAi knockdown of PAIP2A had no significant effect on Kif17 mRNA levels. NS, $p \geq 0.05$, two-tailed t test. n = 3 independent experiments.

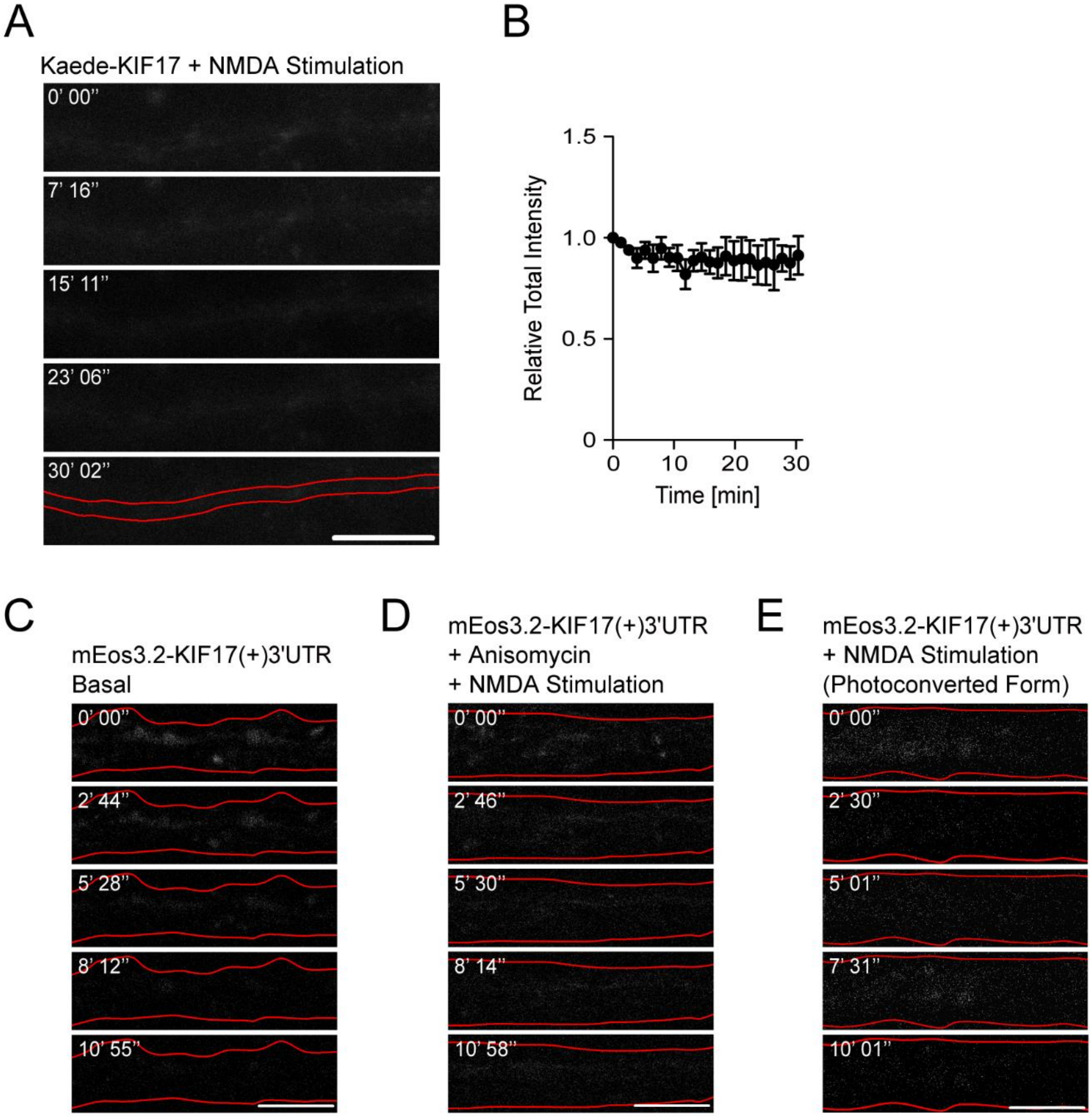
A
Kaede-KIF17 + NMDA Stimulation
0' 00''
7' 16''
15' 11''
23' 06''
30' 02''
B
1.5
1.0
0.5
0
Relative Total Intensity
0
10
20
30
Time [min]
C
mEos3.2-KIF17(+)3'UTR
Basal
0' 00''
2' 44''
5' 28''
8' 12''
10' 55''
D
mEos3.2-KIF17(+)3'UTR
+ Anisomycin
+ NMDA Stimulation
0' 00''
2' 46''
5' 30''
8' 14''
10' 58''
E
mEos3.2-KIF17(+)3'UTR
+ NMDA Stimulation
(Photoconverted Form)
0' 00''
2' 30''
5' 01''
7' 31''
10' 01''

Fig. S8, related to Fig. 6. Supplemental experiments related to the induction of local KIF17 synthesis at the dendritic shaft. (A and **B)** Stimulation with NMDA did not induce the *Kif17* coding region-mediated local synthesis of the translation reporter Kaede and KIF17. Hippocampal neurons (DIV 21–24) transduced with a lentiviral construct containing Kaede fused to KIF17 (Kaede-KIF17) were stimulated with NMDA after photoconversion of Kaede. Kaede-KIF17 puncta were not synthesized (A). The total relative intensity did not change significantly (B). Red lines trace the morphology of the dendrites. Scale bar, 10 μm. $p \geq 0.05$, one-way ANOVA. n = 4 dendrites from three independent cultures. **(C** and **D)** Time-lapse images of dendrites transfected with mEos3.2-KIF17(+)3'UTR. mEos3.2-KIF17(+) puncta were not synthesized throughout the observation without stimulation (C) or with stimulation in the presence of anisomycin (D). Scale bar, 5μm. **(E)** Time-lapse images demonstrating the photoconverted form of mEos3.2-KIF17(+) upon NMDA stimulation. The original form of mEos3.2-KIF17(+) is represented in Fig. 6A. Scale bar, 5 μm.

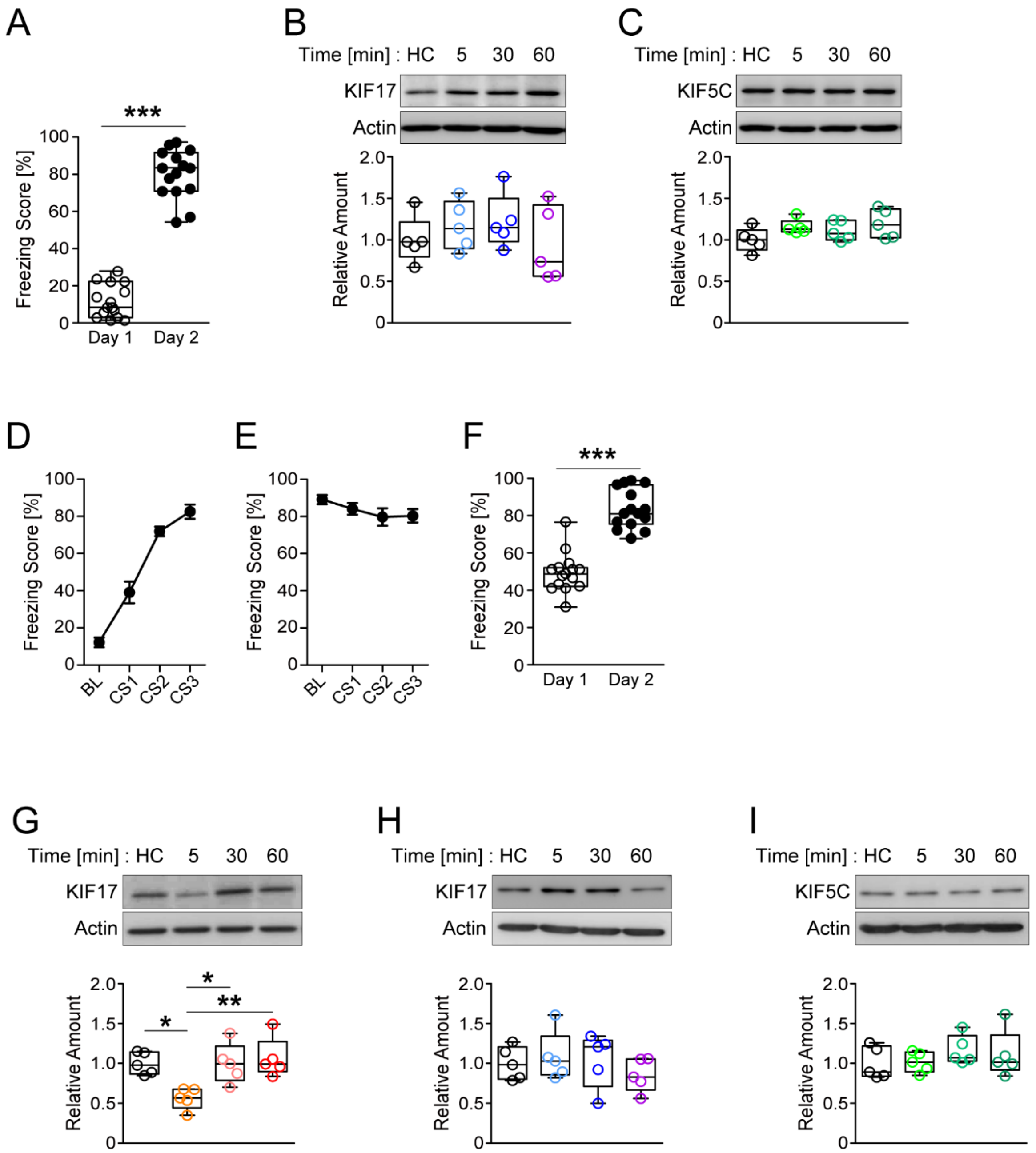
A
B
C
D
E
F
G
H
I
Freezing Score [%]
Day 1
Day 2
***
Time [min] : HC 5 30 60
KIF17
KIF5C
Actin
Relative Amount
BL
CS1
CS2
CS3
*
**

Fig. S9, related to Fig. 7. Supplemental experiments for *in vivo* analyses. (A) Freezing scores of the contextual fear conditioning test. The freezing score of day 2 was significantly higher than that of day 1. In box plots, the central line represents the median, the edges of the box represent the interquartile range, and whiskers represent the minimum to the maximum. ***$p < 0.001$, two-tailed t test. n = 15 mice. **(B** and **C)** Time courses of KIF17 expression in the mouse mPFC (B) and of KIF5C expression in the mouse hippocampus (C) after contextual fear memory retrieval. Neither of them changed significantly. $p \geq 0.05$, one-way ANOVA. n = 5 mice groups. **(D** and **E)** Freezing scores of the cued fear conditioning test. The freezing score during memory acquisition increased continuously (D). The freezing score during memory retrieval was unchanged but remained high throughout the trials (E). BL, baseline; CS, conditioned stimulus. n = 15 mice. **(F)** Total freezing scores of the cued fear conditioning test. The freezing score of day 2 was significantly higher than that of day 1. ***$p < 0.001$, two-tailed t test. n = 15 mice. **(G)** Time course of KIF17 expression in the mouse hippocampus after cued fear memory retrieval. KIF17 expression was downregulated within 5 min and subsequently upregulated to baseline. $p < 0.01$, one-way ANOVA; *$p < 0.05$, **$p < 0.01$, Bonferroni's post hoc comparison. n = 5 mice groups. **(H** and **I)** Time courses of KIF17 expression in the mouse mPFC (H) and of KIF5C expression in the mouse hippocampus (I) after cued fear memory retrieval. Neither of them changed significantly. $p \geq 0.05$, one-way ANOVA. n = 5 mice groups.

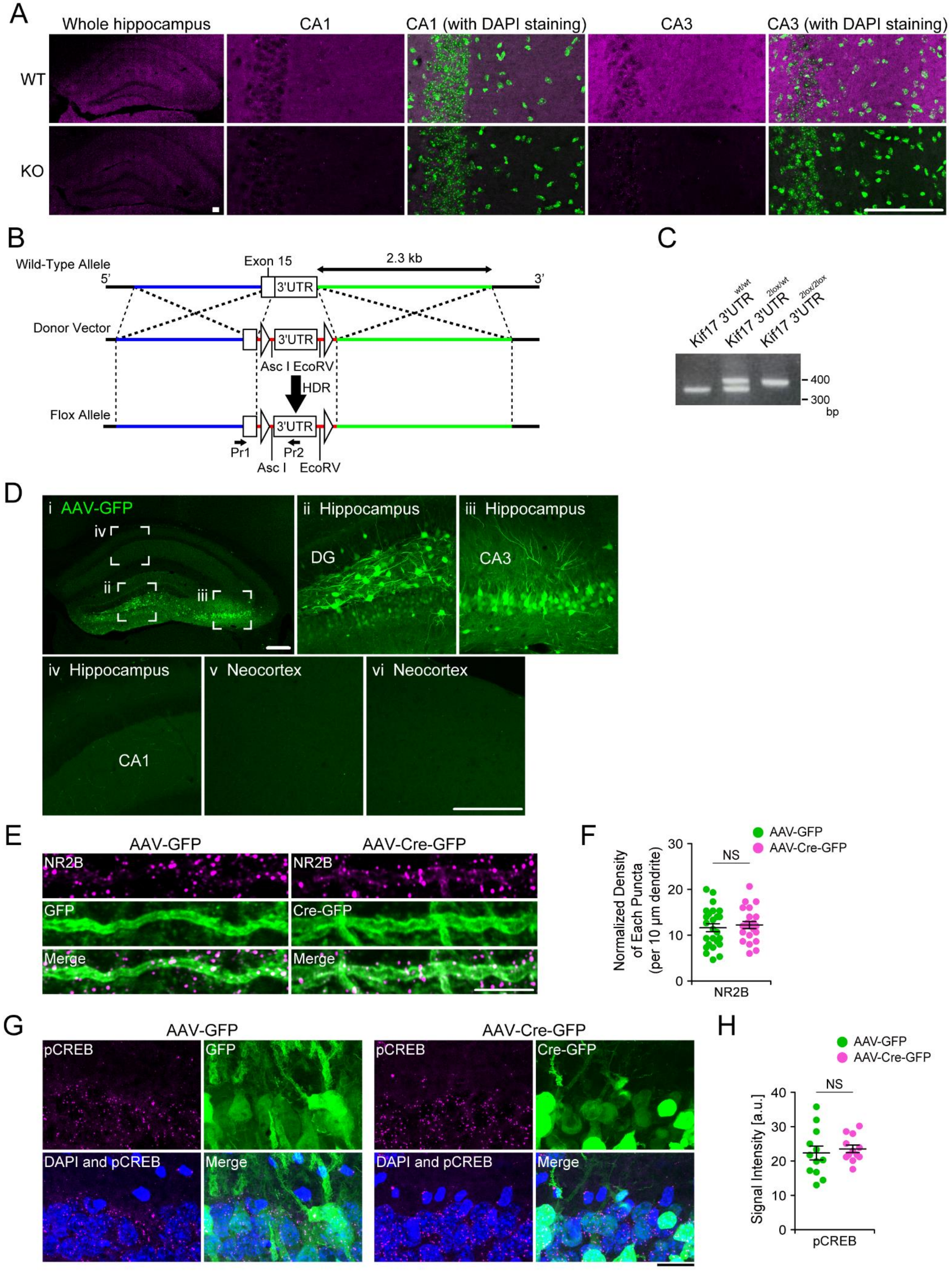

A
Whole hippocampus
CA1
CA1 (with DAPI staining)
CA3
CA3 (with DAPI staining)
WT
KO
B
Wild-Type Allele
Exon 15
2.3 kb
5'
3'UTR
3'
Donor Vector
Asc I EcoRV
HDR
Flox Allele
Pr1
Pr2
Asc I
EcoRV
C
Kif17 3'UTR wt/wt
Kif17 3'UTR 2lox/wt
Kif17 3'UTR 2lox/2lox
400
300
bp
D
i AAV-GFP
iv
ii
iii
ii Hippocampus
DG
iii Hippocampus
CA3
iv Hippocampus
CA1
v Neocortex
vi Neocortex
E
AAV-GFP
AAV-Cre-GFP
NR2B
GFP
Cre-GFP
Merge
F
Normalized Density of Each Puncta (per 10 μm dendrite)
AAV-GFP
AAV-Cre-GFP
NS
NR2B
0
10
20
30
G
AAV-GFP
AAV-Cre-GFP
pCREB
GFP
Cre-GFP
DAPI and pCREB
Merge
H
AAV-GFP
AAV-Cre-GFP
NS
Signal Intensity [a.u.]
pCREB
0
10
20
30
40

Fig. S10, related to Fig. 7 and 8. Supplemental experiments related to the conditional deletion of *Kif17* 3'UTR by the hippocampus-specific transduction of AAV vectors. (A) *In situ* hybridizations in the WT (*kif17*$^{+/+}$) and KO (*kif17*$^{-/-}$) mouse hippocampus of coronal brain sections performed with quantitative FISH probe to detect Kif17 mRNA at lower (most left panel) and higher (the other panels) magnifications. DAPI staining was concomitantly performed. Tile scanning with an LSM780 confocal laser-scanning microscope was used for the lower magnification images. Scale bars, 100 µm. **(B)** Gene targeting strategy used to generate the *Kif17* 3'UTR floxed mice. *loxP* sequences were inserted using the CRISPR/Cas9 system. Open arrowheads, *loxP* sequences; HDR, homology directed repair; Pr, primer. **(C)** Genomic PCR of DNA from the mouse tails of the indicated genotypes amplified by the PCR primers Pr1 and Pr2 in (B). **(D)** Representative images of AAV-GFP expressing cells at lower (i) and higher (ii–vi) magnification views of the hippocampus (ii–iv) and deep (v) and superficial (vi) layers of the neocortex resulting from bilateral hippocampus-specific injection of AAV-GFP. Boxes in (i) represent magnified regions in the following panels. Tile scanning with an LSM780 confocal laser-scanning microscope was used for the lower magnification image (i). Scale bars, 200 µm. **(E and F)** Representative images of immunofluorescence histochemistry demonstrating NR2B expression in AAV-infected dendrites at CA3 region of coronal brain sections (E), and the quantification of the normalized density of anti-NR2B positive puncta in dendrites (F). Scale bar, 10 µm. NS, $p \geq 0.05$, two-tailed *t* test. n = 25 (AAV-GFP) and 22 (AAV-Cre-GFP) dendrites from three mice pairs. **(G and H)** Representative images of immunofluorescence histochemistry demonstrating CREB phosphorylation in AAV-infected neurons at CA3 region of coronal brain sections (G) and the quantification of the signal intensity of anti-phospho-CREB positive puncta in somata. DAPI staining was concomitantly performed. Scale bar, 20 µm. NS, $p \geq 0.05$, two-tailed *t* test. n = 12 brain slices from three mice pairs.

Movies

Movie S1, related to Fig. 2. Degradation of moving PA-GFP-KIF17 in dendrites upon NMDA stimulation.

Live imaging of hippocampal neuron dendrites expressing PA-GFP-KIF17 without (upper) and with (lower) NMDA stimulation. The dendrites were monitored for 1.63 min using an LSM780 confocal laser-scanning microscope (Zeiss). Note that PA-GFP-KIF17 moved into the observed area and disappeared by moving through the area (upper) or by vanishing in the area (lower). Related to Fig. 2E. Scale bar, 5 μm.

Movie S2, related to Fig. 4. Significance of Kaede synthesis driven by KIF17 3'UTR in a neuron upon NMDA stimulation.

Live imaging of hippocampal neurons transfected with Kaede-KIF17(-)3'UTR without (left) and upon (right) NMDA stimulation. The movies were taken over the course of 68.1 min using an LSM780 confocal laser-scanning microscope. Note that Kaede fluorescence appeared in dendrites and soma upon NMDA stimulation (right) but not without NMDA stimulation (left). The circles represent the measured area in Fig. 4, B and D. Related to Fig. 4, A and C. Scale bar, 20 μm.

Movie S3, related to Fig. 4. Significance of Kaede synthesis driven by KIF17 3'UTR in isolated dendrites upon NMDA stimulation.

Live imaging of isolated dendrites transfected with Kaede-KIF17(-)3'UTR upon NMDA stimulation. The movie was taken over the course of 60.9 min using a LSM780 confocal laser-scanning microscope. Note that Kaede proteins synthesized in isolated dendrites did not diffuse into the bleached region because of their aggregations. The green line rectangle represents the bleached region. Related to Fig. 4F. Scale bar, 20 μm.

Movie S4, related to Fig. 4. Significance of local Kaede synthesis driven by KIF17 3'UTR at dendritic shafts upon NMDA stimulation.

High-magnification live imaging of hippocampal neuron dendrites transfected with Kaede-

KIF17(-)3'UTR upon NMDA stimulation. The movie was taken over the course of 59.8 min using a LSM780 confocal laser-scanning microscope. Note that the arrow indicates where locally synthesized Kaede puncta aggregated and were suspended. Red lines trace the morphology of the dendrites. Related to Fig. 4H. Scale bar, 10 µm.

Movie S5, related to Fig. 5. Pharmacological analysis of local Kaede synthesis driven by KIF17 3'UTR upon NMDA stimulation.

Live imaging after NMDA stimulation of hippocampal neuron dendrites transfected with Kaede-KIF17(-)3'UTR and pretreated with each antagonist. The movies were taken over the course of 62.0 min using an LSM780 confocal laser-scanning microscope. Note that individual pretreatment with anisomycin (top) and calpeptin (bottom) attenuated local synthesis of Kaede but not pretreatment with actinomycin D (second) and lactacystin A (third). The circles represent the measured area in Fig. 5B. Related to Fig. 5A. Scale bar, 20 µm.

Movie S6, related to Fig. 5. Attenuation of local Kaede synthesis driven by KIF17 3'UTR in NMDA-stimulated dendrites after RNAi knockdown of PAIP2A.

Live imaging of NMDA-stimulated dendrites transfected with control shRNA (left) and shRNA against PAIP2A (right), in addition to Kaede-KIF17(-)3'UTR. The movies were taken over the course of 64.9 min using an LSM780 confocal laser-scanning microscope. Note that local synthesis of Kaede was attenuated in dendrites transfected with shRNA against PAIP2A (right). The circles represent the measured area in Fig. 5E. Related to Fig. 5D. Scale bar, 10 µm.

Movie S7, related to Fig. 6. Motility of the newly synthesized mEos3.2-KIF17 in dendritic shafts.

Live imaging of hippocampal neuron dendrites transfected with mEos3.2-KIF17(+)3'UTR without (top) and upon (second) NMDA stimulation, and that of dendrites transfected with mEos3.2-KIF17(ΔM)3'UTR (third) and mEos3.2-KIF17(ΔT)3'UTR (bottom) upon NMDA

stimulation. The dendrites were monitored for 2.23 min using an LSM780 confocal laser-scanning microscope. Note that mEos3.2-KIF17(+) and mEos3.2-KIF17(ΔT) synthesized and moved along the dendrites (second and bottom), while mEos3.2-KIF17(ΔM) synthesized but did not move along the dendrite (third). Without stimulation, mEos3.2-KIF17(+) was not synthesized throughout the observation (top). Red lines trace the morphology of the dendrites. Related to Fig. 6A to C, and fig. S8C. Scale bar, 5 μm.
